# A hybrid convolutional-transformer neural network model for photovoltaic fault detection and localization

**DOI:** 10.1038/s41598-026-57859-7

**Published:** 2026-07-24

**Authors:** Ebrahim A. Ramadan, Nada M. Moawad, Belal A. Abouzalam, Wessam F. Abouzaid, Ghada M. El-Banby

**Affiliations:** 1 Department of Industrial Electronics and Control Engineering, Faculty of Electronic Engineering, Menoufia University, Menouf, 32952 Egypt; 2 Department of Electrical Engineering of Computer and Control Systems, Faculty of Engineering, Kafrelsheikh University, Kafrelsheikh, 33516 Egypt

**Keywords:** Energy science and technology, Engineering, Mathematics and computing

## Abstract

Energy retention from losses is the primary goal of fault detection methodology for photovoltaic (PV) solar systems. A fault detection model should be designed effectively to minimize power and cost waste. We propose a novel fault detection and localization method that leverages deep learning techniques for PV systems. The model is a hybrid semantic segmentation method that combines Convolutional Neural Networks (CNN) and multi-level transformer neural networks. We examine our model on four different segmentation datasets, which have varied characteristics and different capturing conditions, to ensure our model generalization. Using aerial inspection, the first thermal dataset images give a high performance in locating the faulty cells in PV arrays with an accuracy and global precision of 99.89%, a mean average precision (mAP) of 88.73%, a mean intersection over union (mIoU) of 76.29%, and 84.48% of mean dice (mDice, or mean/unweighted F1 score). We use three electroluminescence datasets to investigate the precise location and class of different fault types and minor cracks at the cell level. The second dataset result achieved perfect detection and segmentation of anomaly areas in cells with 99% accuracy, 96.36% mIoU, 98.13% mDice, and 98.41% mAP. The first two datasets contain binary segmentation images with fault and no-fault classes; to accommodate multiple classes, we have utilized the third and fourth datasets. The third dataset, comprising five PV failure classes, is evaluated against other models, yielding a superior performance of 96.27% precision, 77.64% mAP, 57.3% mIoU, and 68.45% mDice. The final dataset has 29 segmentation classes for testing 25 classes, for which it achieves a precision of 95.05%, 66.7% mAP, 49.3% mIoU, and 59.3% mDice.

## Introduction

Renewable energy resources have grown increasingly vital recently, as they mitigate environmental degradation. One of the most crucial resources is solar energy, which is expected to overtake the most common renewable energy sources, such as wind and hydropower, by the year 2030^1^. It is also anticipated to supplant traditional resources (e.g., natural gas and oil) in the foreseeable future due to its affordability, safety, and cleanliness. More than 1.64 TW of photovoltaic plants have been installed to produce solar energy in 2023, with a generated capacity of approximately 1.294 TWh, while over 50% were installed in the past three years. PV is the fastest-growing renewable energy source, with a year-over-year increase of more than one quarter (25.6%), resulting in over 35 countries having a gigawatt-scale annual market^2,3^. PV systems encounter numerous energy losses due to multiple defects. Particular strategies must be implemented to prevent or mitigate losses, depending upon the severity and cause of the defects. Temporary failures, such as soiling and shading, can be readily addressed; however, permanent ones, including cracks and delamination, may necessitate professional attention^4^. Consequently, a robust fault detection and diagnosis system (FDD) is necessary for monitoring photovoltaic (PV) systems to enhance their performance and dependability by promptly identifying and locating failures as they occur. The FDD system should detect a fault, identify its type and exact location and promptly isolate it to prevent the risk of fire in the event of serious failures. This process of isolation is exceedingly difficult and necessitates additional information and specialized expertise from Operation and Maintenance (O&M) services, who determine whether the fault poses a threat and if immediate intervention is necessary^5^.

Artificial intelligence (AI) technologies are involved in a significant number of aspects and fields that facilitate dealing with hard or complex problems that help the machine to act like humans and see what they see, especially in computer vision (CV) applications that would assist fault detection systems for automatic supervision and monitoring of PV systems. FDD systems based on AI methods such as deep learning (DL) and machine learning (ML) can be designed using historical data-driven information from actual PV systems. Other model-based FDD systems depend on the mathematical principles of the PV system or model simulation, which compares readings from sensors with normal operation to find out the problem but not its precise location. DL has become an effective tool for diagnosing and detecting faults in PV modules rather than traditional techniques that may be insufficient, particularly for large-scale PV arrays^6^, because DL can adapt to new knowledge and handle unpredictable conditions, making it effective for lengthy and complex tasks as the linkages between inputs and outputs become clear.

Computer vision techniques enable computers to analyze, interpret, and process the visual world through the use of artificial intelligence and deep learning. One common method for detecting faults in solar modules is visual and thermal imaging technologies. Thus, a data-driven operating FDD system depends on collecting a large number of images under many situations and various scenarios to give the machine the opportunity to learn. In the thermography (infrared IR) technique, the images are collected remotely, likely by drones to cover big areas, without any intervention or system interruption as one of the nondestructive methods. It is a quick, straightforward, dependable, precise, and cost-effective method that requires just an infrared camera to distribute the distinctive properties of PV modules in two dimensions.

One of the recent deep learning techniques, the transformer neural network (TNN), was originally developed for natural language processing (NLP)^7^. It was considered a revolution in sequential processing since it could handle complex dependencies for large tasks employing attention mechanisms by processing sequential input in parallel. In 2020, the authors of the vision transformer model^8^ changed the transformer NN’s design to make it more practical for computer vision applications such as image recognition and classification procedures. Since then, Transformer NN models have been widely adopted in numerous fields, such as autonomous driving, medical image analysis, remote sensing, land coverage analysis and recently PV fault detection^9–15^.

The FDD system of^9^ uses a new deep learning model based on a modified multiscale Vision Transformer (ViT) neural network technique to improve the globality and robustness of the system. Preprocessing methodologies are applied to enhance and increase 20,000 thermography images of the dataset for detecting and classifying eleven PV module anomalies, e.g., soiling, bypass diode, cracking, shadowing, and hotspots.

Also, in^10^, a Vision Transformer model and five hybrid ViT models, which combine the ViT model with different kinds of Machine Learning (ML) algorithms, are the methods that were developed and compared in this study to handle the problem of classifying and comprehending the nature of faults in thermal images.

To facilitate dynamic communication and data exchange between dispersed energy resources, such as PV systems, and the grid infrastructure, smart grids and Internet of Things technologies (IoT) are essential. Real-time monitoring, adaptive load management, and predictive defect detection are all made possible by IoT-enabled smart grids, which improve grid reliability. The work by^16^ identifies PV system problems using PV arrays’ on-board devices (IoT modules) that apply a fault detection algorithm based on traditional I-V curve electrical measurement analysis and then notify a number of unmanned aerial vehicles (UAVs) with RGB and infrared cameras to do thermal and visual examinations of the damaged photovoltaic panels. The IoT modules receive the inspection data and take the necessary action.

One of the key deep learning tasks in computer vision is the semantic segmentation method, which assigns a class (label) to each pixel in an image rather than classifying the entire image (classification) or just a portion of it (object recognition). The model finally produces a color copy of the original image based on the PV defects classification. This algorithm can manage the diverse, intricate backgrounds and textures of PV panel surfaces, precisely delineating distinct parts of the panels from images and recognizing the location and kind of failures. The DeepLab series, Mask R-CNN, PSPNet, SegNet, and U-Net are some examples of popular semantic segmentation techniques.

The method in^17^ introduced a segmentation methodology in which thermal images are collected from a UAV fitted with infrared sensors to identify faulty panels on large solar plants. Segmentation models with varying encoder architectures are applied, which include DeepLabV3+, Feature Pyramid Network (FPN), and U-Net. However, it just identifies the module’s cells as either faulty or non-faulty.

The study by^18^ presents a deep learning framework that integrates Attention Mechanisms, Residual Blocks, and Atrous Spatial Pyramid Pooling (ASPP) to improve the U-Net architecture after the image preprocessing stage for IR images. Together, these improvements enhance contextual comprehension, fault localization, and feature extraction, thereby overcoming the limitations of conventional segmentation techniques.

On the other hand, electroluminescence (EL) imaging technologies are needed to overcome the drawbacks of low resolution in RGB and thermal images and also the challenge of detecting small (often invisible) defects. EL imaging is an essential diagnostic technique for evaluating the quality and functionality of PV modules. In this technique, PV modules are provided with direct current (DC) to facilitate radiative recombination in the solar cells during the capturing of the images. Numerous defects, including cracks, finger interruptions, delamination, and ablation, can be seen in these images; each one could require a different solution for repairing or replacing it.

Authors of^19^ implement a semantic segmentation model for EL images to detect irregularities in photovoltaic panels at the cell level using Convolutional Block Attention Module (CBAM), Attention Refinement Module (ARM), and ASPP modules and employ K-Net as a baseline to improve network performance, especially in identifying small-area connected faults and defect edges.

Another study^20^ is able to distinguish between cracks, contact interruptions, cell connection failures, and contact corrosion for both multi-crystalline and monocrystalline silicon cells. The suggested model makes use of a DeepLabv3 segmentation model with a ResNet-50 backbone. In order to address class imbalance, it was trained using 17,064 EL pictures, including 256 physically realistic simulated images of PV cells.

Based on EL polarization imaging and the fundamental mechanism of PV cell crack generation, the work in^21^ suggests a new semantic segmentation method for PV cells. Three single-channel pictures of PV cells are obtained, which are polarization intensity (I), degree of polarization (DOP), and polarization quadrature (Q), which are then stacked into several channels to produce an extensive dataset. After the dataset has been annotated for training purposes, the texture features linked to microcrack faults are analyzed. In order to optimize the utilization of the unique characteristics of the polarized three-channel images, they improve the conventional U-Net design by incorporating the Efficient Channel Attention module, depth-wise separable convolutions, and the Squeeze-and-Excitation attention mechanism.

A new architecture has been developed^22^ for semantically segmenting 29 different characteristics and failures in PV panel EL pictures by replacing the SegNet architecture encoder with a pre-trained VGG16 encoder and using a CBAM block to improve the decoder’s capacity for producing fine-grained segmentations.

Also, a ViT SegFormer-based PV cell segmentation system for fault identification is employed^11^ to automate the visual examination of defects in photovoltaic modules of EL images, incorporating fault pseudo-colorization.

In order to address the problem of extreme imbalance between abnormal and background pixel distributions, the authors of^23^ presented a method for detecting micro-crack anomalies in photovoltaic modules based on an M-shape structure and attention module for classification and segmentation network. They showed superior performance for segmenting micro-cracks and more effectively extracted and fused shallow-level and deep-level features in the segmentation network.

In^24^ they present an end-to-end deep learning pipeline that uses EL pictures to identify, locate, and segment cell-level anomalies from complete solar modules using weakly supervised segmentation (autoencoder), image classification (EfficientNet), and object detection (modified Faster-RNN).

In the field of industrial defect detection, there are also promising technologies using model optimization, attention mechanisms, and data augmentation. The YOLOv5-based lightweight algorithm in^25^ prioritizes computational efficiency and multi-class defect recognition, making it suitable for edge deployment (resource-constrained). In contrast, the MSWindD-YOLO model in^26^ specifically enhances feature discrimination by integrating attention mechanisms to capture subtle or irregular surface defects better. Meanwhile, the EP-YOLOv12n in^27^ with improved ant colony optimization uniquely couples adaptive defect detection with optimized inspection path planning, addressing both perception and navigation challenges in real-world environments.

Recent literature has emphasized lightweight architectures for solar PV defect detection under edge deployment constraints. For object detection, in^28^, they use a Deep Convolutional Generative Adversarial Network (DCGAN)-based minority class augmentation coupled with lightweight YOLO variants, which have been proposed for edge-deployable PV fault localization. Similarly, the model in^29^ of YOLOv8n-GBE integrates ghost convolutions with BiFPN-ECA attention to reduce parameters while maintaining localization accuracy. For the classification task, the authors in^30^ depend on FPGA-orientated designs using patch-wise reusable CNN IP cores, which have demonstrated high-speed PV module defect classification. In^31^, the model of AAPN-Tiny offers a compact adaptive attention pyramid architecture specifically for multi-class fault diagnosis on edge devices, while in^32^, pruned and low-rank-optimized tiny residual architectures have been tailored for Edge TPU acceleration. For segmentation of faults, the model in^33^ utilizes a lightweight SegFormer architecture featuring a modified Mix Transformer encoder and an optimized Multi-Layer Perceptron decoder. These works priorities inference speed, power efficiency, and model compactness, critical for large-scale or real-time deployments.

### Motivations and contributions

Traditional FDD methods, which include continuous systems monitoring using various statistical analyses, require significant effort to achieve acceptable results for finding PV failures. It may identify the failed element or module, but it does not specify the affected regions, types of cracks, or the exact defective parts of the cells. Semantic segmentation as one of the FDD methods is designed to locate the affected parts of PV elements, enabling efficient handling and maintenance. Therefore, it is imperative to develop efficient methodologies to assess the health state of photovoltaic plants by detecting defects, determining their type and location and predicting failure patterns of potentially affected combining usage components^34^. To rapidly and effectively identify, locate, and classify solar PV system faults’ exact location automatically, we suggest a reliable FDD system built on a hybrid transformer neural network that is applied for both thermal and EL imaging to ensure the system’s robustness under various imaging and capturing conditions. We aim to achieve this goal by combining IR image segmentation that is acquired contactlessly by UAVs to identify the impacted cells in large PV systems, followed by a detailed examination of microcracks and minor defects through EL image semantic segmentation, which identified the precise location and classified fault regions in the cell. Our main contributions in this work can be summarized as follows:

- A novel automated fault detection and diagnosis (FDD) system is proposed, built upon a hybrid semantic segmentation framework that integrates Convolutional Neural Networks (CNNs) with multi-level Transformer Networks. This hybridization leverages the strengths of both architectures: CNNs effectively capture local spatial features with fewer layers, while Transformers, through their attention mechanisms, learn global contextual relationships across the entire image to accurately associate class types with their corresponding locations.
- An enhanced U-Net architecture is developed by embedding multi-scale Transformer layers within the encoder. This modification enables more comprehensive multi-level feature extraction, substantially improving the classical U-Net’s segmentation precision and reliability. Consequently, the proposed PV FDD system achieves more accurate fault localization and classification, contributing to enhanced solar system protection and reduced energy and maintenance costs.
- Extensive validation and evaluation of the proposed PV FDD model are conducted using diverse photovoltaic datasets, including one thermal dataset and three electroluminescence (EL) datasets. To the best of our knowledge, such a comprehensive cross-dataset evaluation has not been reported previously. Comparative results with several state-of-the-art models, including published articles, demonstrate the superior segmentation accuracy and robustness of the proposed approach in both binary and multi-class fault scenarios.

The remainder of this paper is structured as follows: Sect. 2 introduces materials and methodology. Section 3 presents the training process, evaluation results, and comparative analysis with other state-of-the-art models. Finally, Sect. 4 provides the conclusions and outlines potential directions for future work.

## Materials and methodology

To comprehensively evaluate the robustness and generalization capability of the proposed hybrid CNN–Transformer fault detection and localization framework, four distinct photovoltaic (PV) datasets were employed, encompassing both thermal and electroluminescence (EL) imaging modalities. Each dataset represents a distinct imaging condition, defect type, and level of segmentation complexity.

## Thermal dataset (Photovoltaic thermal images)

**Fig. 1:**
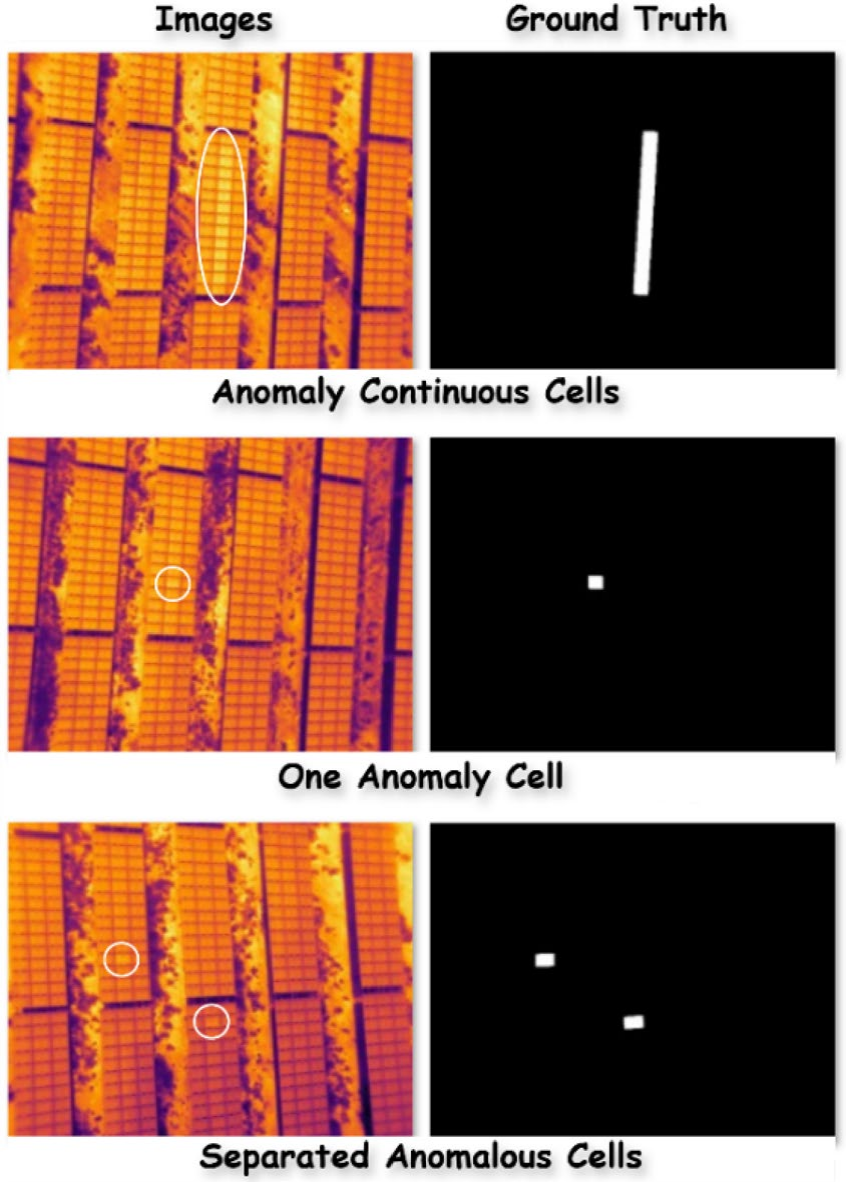
First PV thermal dataset samples.

This dataset consists of 1,009 infrared thermographic images^35^ captured by a UAV-mounted *Flir Tau 2 640* thermal camera with a resolution of 640 × 512 pixels during the inspection of a 66 MW PV plant located in *Tombourke*,* South Africa*^36^. Each image is paired with a binary ground truth mask indicating faulty and non-faulty cells, as shown in Fig. 1. The dataset was split into 70% training, 20% validation, and 10% testing subsets. It serves to evaluate the system’s ability to detect defective cells at the module level under large-scale, real-field conditions.

## PVEL-S dataset (Electroluminescence images)

**Fig. 2:**
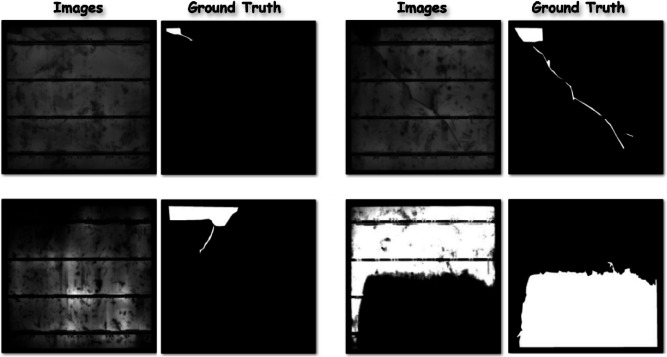
Second PV electroluminescence (PVEL-S) dataset samples.

The PVEL-S dataset comprises 1,200 EL images captured by a *1024 × 1024 cooled CCD camera (WP-US146)* in a darkroom under 24 V DC and 8 A excitation current^37^. The dataset includes twelve types of micro-defects, such as grid cracks, black cores, and thick lines^38^, grouped into two classes: *Defective* and *Non-defective*^19^, as illustrated in Fig. 2. Images were divided into 70% for training and 30% for validation. This dataset is utilized to assess fine-grained fault localization at the cell level.

## UCF-EL dataset

**Fig. 3:**
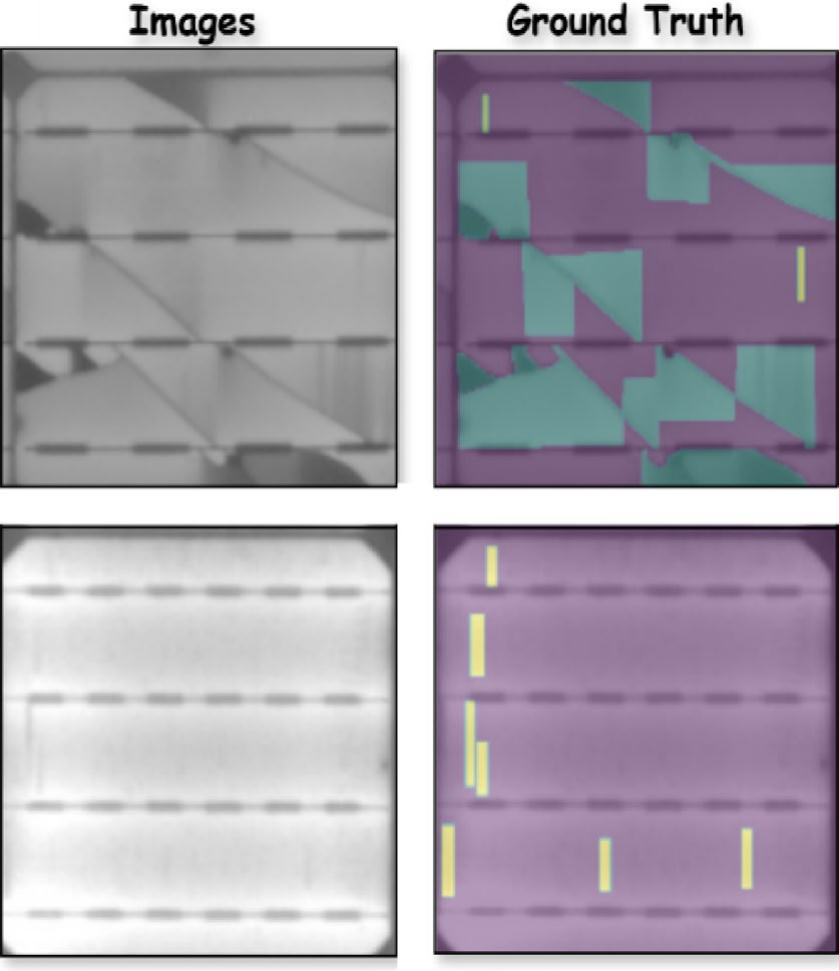
Third PV electroluminescence (UCF-EL) dataset samples.

Provided by the *University of Central Florida Photovoltaics Lab*, this dataset contains 11,851 EL images (are used from total 17,064), including both real and simulated images of crystalline silicon (c-Si) PV cells^39^. It originally included nine defect types (e.g., corrosion, cracks, and interconnect failures), which were merged into five main categories (including the no-fault class) to address data imbalance. Ground-truth masks were annotated using the *VGG Image Annotator*^20^; some images are shown in Fig. 3. The dataset was divided into 80% training, 10% validation and 10% testing subsets. It is used to evaluate the model’s multi-class segmentation capability.

## Benchmark EL dataset

**Fig. 4:**
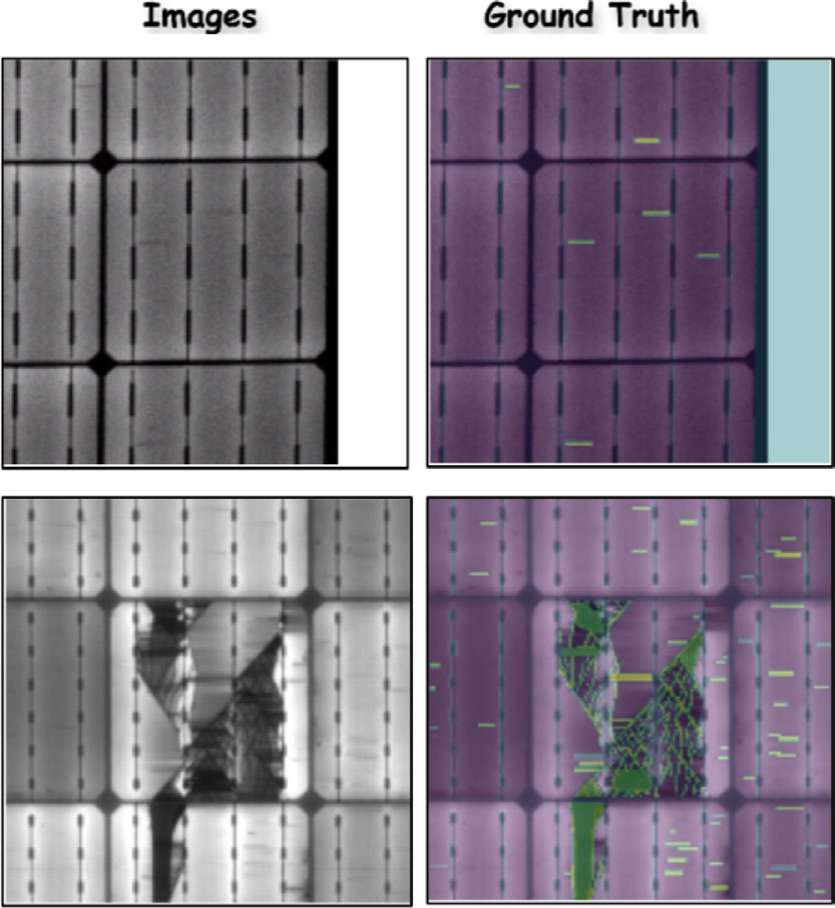
Fourth PV electroluminescence (Benchmark EL) dataset samples.

This dataset integrates **2**,354 EL images collected from five different public and private sources, representing both mono- and multi-crystalline PV modules^40^. It contains 29 segmentation classes, including 16 defect-related classes (e.g., cracks, corrosion, inactive areas) and 13 structural or contextual classes (e.g., busbars, ribbons, frame edges) in Fig. 4. Ground-truth masks were created using the *GNU Image Annotator*, and data augmentation was applied as described in previous works^41,42^. The dataset is divided into 2212 training, 70 validation, and 72 testing images, serving as a challenging benchmark for large-scale multi-class PV defect detection and localization.

Proposed Hybrid Multi-Scale Transformer U-Net Model.

Photovoltaic (PV) systems are susceptible to various failures that can significantly impact performance and overall efficiency post-installation. Continuous monitoring and maintenance are therefore essential to minimize potential faults and performance degradation. However, merely detecting the presence of a fault is insufficient; it is equally important to accurately localize the defect and identify its shape and type.

Semantic segmentation is crucial in deep learning-based fault detection and diagnosis (FDD) systems for PV applications, as it enables pixel-level localization of defects without interfering with system operation. This process produces an annotated version of the original PV image, clearly indicating the faulty regions.

**Fig. 5:**
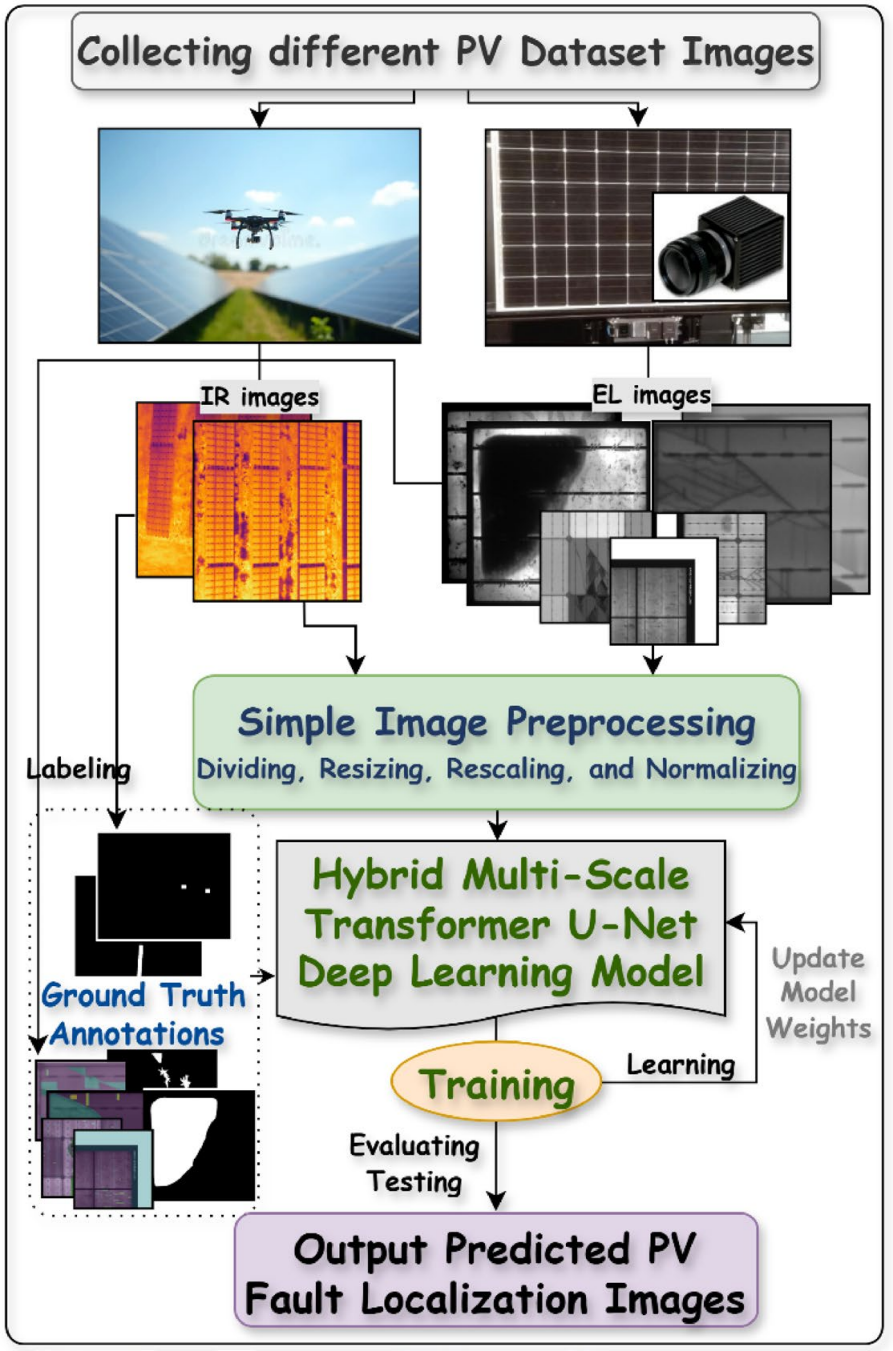
Overall workflow of the proposed PV fault localization framework.

As illustrated in Fig. 5, the proposed methodology starts by collecting diverse PV image datasets containing annotated anomalies. These datasets are then used to train the proposed hybrid multi-scale Transformer-U-Net model, which performs automatic segmentation and localization of PV faults. The model is trained without data enhancement or augmentation to objectively evaluate its inherent performance and robustness across different imaging techniques.

The proposed architecture is based on the U-Net framework but incorporates multiple Transformer layers within the encoder section to capture both local and global feature representations. This hybrid design leverages the spatial feature extraction ability of convolutional neural networks (CNNs) along with the long-range dependency modeling capability of Transformers, enabling more accurate and reliable localization of PV faults under various imaging conditions.

**Fig. 6:**
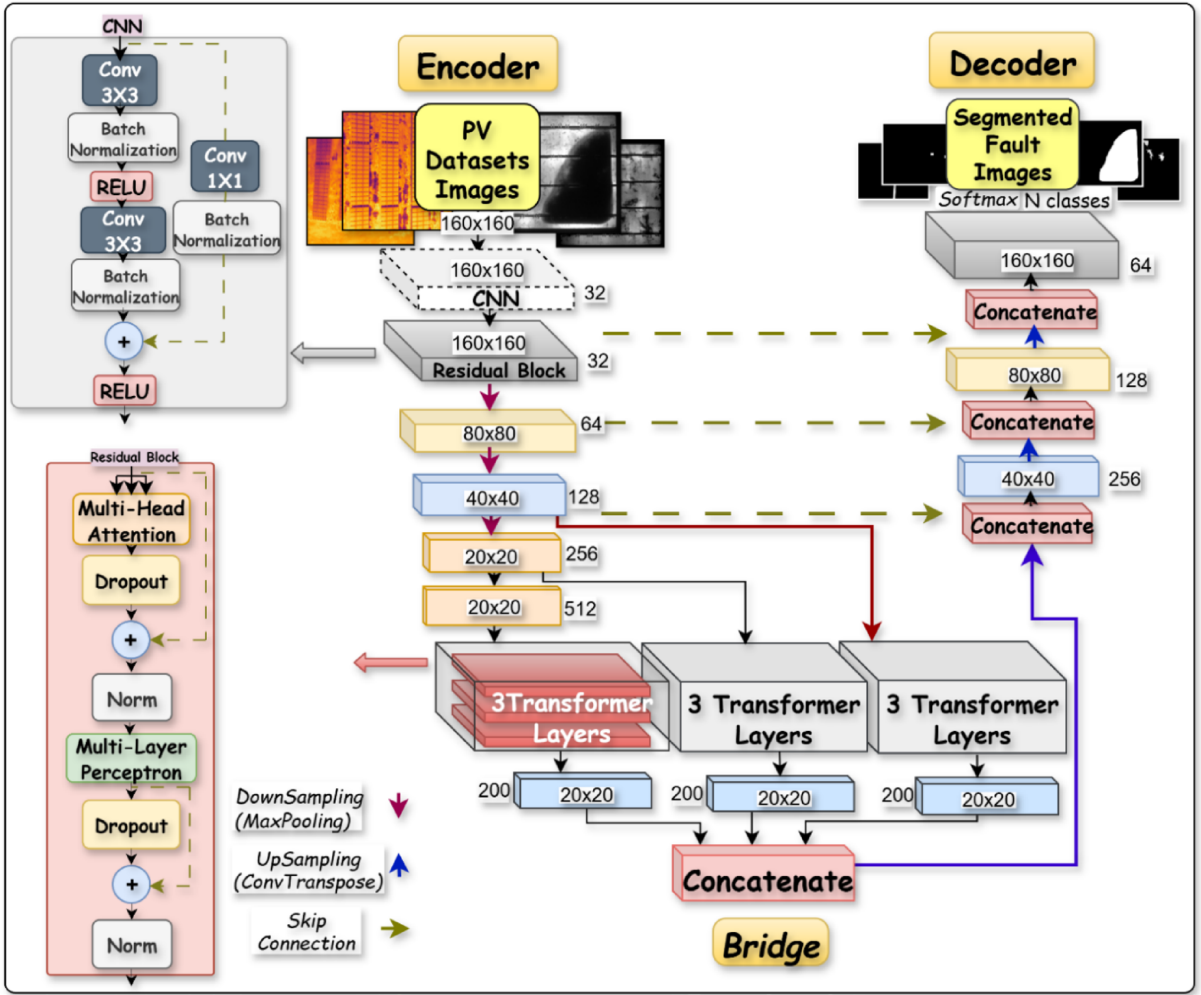
Detailed framework of the proposed hybrid deep learning model.

The detailed framework of the proposed hybrid deep learning model based on an encoder-decoder U-Net architecture^43^ is shown in Fig. 6, which combines convolutional NNs with transformer NNs.

In our model, the cascaded CNN layers are connected by skip connections (residual blocks) to preserve low-level spatial information for improving finer segmentation details and to mitigate the vanishing gradient problem. In the encoder stages, it begins with CNN layers that are down-sampled sequentially and then integrated with multi-level transformer layers to extract relations with unique information from the entire image, resulting in enhanced performance compared to the classical U-Net. The model starts with one convolution NN layer that applies 3 × 3 filters on the dataset images and produces 32 diverse features using the rectified linear unit (ReLU) activation function, followed by the residual block that consists of two 3 × 3 CNN layers separated by batch normalization and ReLU, and also one 1 × 1 CNN that creates a skip connection between the input and the second CNN output (the 1 × 1 CNN is used at down-sampling or up-sampling only; else it is unity), as illustrated in Fig. 6 at the top left. Equation 1 shows the output of the residual block *y*, and *x* denotes its input that resulted from the previous layer^44^.

1 $$\:y={W}_{2}\sigma\:\left({W}_{1}x\right)+{W}_{s}x$$

where *σ* denotes the RELU activation function, *W*_*1*_ and *W*_*2*_ denote the weight matrices of the two cascaded CNN layers, and *W*_*s*_ is a square matrix for the 1 × 1 CNN layer.

The self-attention mechanism in the transformer NN identifies intricate patterns and long-range dependencies due to its global perspective. The multi-head attention mechanism processes data in parallel, allowing them to capture information from various areas of the images and establish a correlation between them. This capability facilitates more accurate predictions through extraction of discriminative features from various areas of the image, enabling effective handling of both local and global features.

By adding three parallel branches of multi-level transformer layers that receive feature images from CNN layers of different depths, the model can preserve the detailed image information and integrate the high-level features of the deep layers with the low-level features of the shallow layers. Each transformer branch comprises three sequential layers with distinct configurations. The main components of the transformer NN layer are detailed on the bottom left side in Fig. 6.

The first branch employs 6 heads in its multi-head self-attention (MHA) layers, and its multi-layer perceptron (MLP) has 128 and 64 dense layers. The second branch layers have 4 heads, with an MLP having 64 and 32 dense layers. The third branch uses 2 heads with MLP dimensions of 32 and 16 and a dropout rate of 0.1 in all transformer layers, along with a normalization layer following each skip connection addition.

The self-attention layer converts the input image features into matrices *V*,* Q*, and *K*. Here, *V* represents the value associated with a query *Q*, which corresponds to an image feature, and *K* denotes the key representing all other image features. This process utilizes a scaled dot product, as shown in Eq. 2, to determine the degree of importance. Equation 3 shows the multi-head layer when concatenating the result of the attentions of all *h* heads.

2 $$\:{head}_{i}=Attention\left(Q,K,V\right)=\:Softmax\left(\frac{Q{K}^{T}}{\sqrt{{d}_{model}}}\right)V$$

3 $$\:MultiHead\left(Q,K,V\right)=Concat\left({head}_{1}\:,\:\dots\:\:,\:{head}_{h}\right){W}^{O}\:$$

where *d*_*model*_ represents the dimension of each head *h*, and$\:\:{W}^{O}\in\:{\mathbb{R}}^{{d}_{model}\times\:{d}_{model}}$. The outputs of the transformer branches are fused again in CNN residual blocks, acting as a bridge and bottleneck that incorporates positional information to the previous transformer layers without the need for a positional encoding embedding at the beginning of the transformer layers, as in the ViT model^11^. Then, the outputs are concatenated to initiate up-sampling in the decoder stages, which conclude with the final CNN layer using the SoftMax activation function to determine the correct pixel class decisions.

The proposed model benefits from merging CNN, which identifies the fine-grained defects, and the transformer NN, which analyzes the entire image to contextualize identified defects within the global scene due to its global contextual information ability, enabling our system to accurately detect, localize, and classify all defect types. Using a CNN with a transformer TNN reduces our model complexity. It eliminates the need for a large model size, substantial image data, or a pre-trained model that pure transformer NNs would require to enhance their precision.

We were inspired by the idea of using residual blocks in the encoder from ResNet18^44^ owing to their high efficiency, ease of implementation, strong generalization capability, fast training with limited parameters (sort of lightweight design), and consistent high accuracy. Our compact model can be helpful for IoT and edge device deployment for online PV fault localization implementation.

The proposed model, in its current configuration, is more suitable for offline high-precision inspection or cloud/edge-server cooperative deployment due to its slightly higher computational complexity and inference time. Real-time edge-only applications require less than about 30 ms latency; a pruned version of our model, then, would be preferred, which may be achieved by removing one Transformer branch and reducing the channels by 1.5, sacrificing approximately 2.5% mIoU to double the speedup. Preliminary profiling on NVIDIA Jetson Orin Nano yields ~ 45 ms per 512 × 512 image (22 FPS), with a model size of 168 MB and peak RAM of ~ 420 MB. While feasible for edge GPU platforms, this exceeds the memory constraints of low-power IoT devices (e.g., ARM Cortex-M class with less than 512 MB of total memory). Future work must therefore explore: quantization-aware training to reduce memory and latency; structured pruning of Transformer heads with minimal accuracy loss; TinyML-friendly architectures (e.g., MobileNet + linear attention); and hardware-software co-design using FPGA or Edge TPU accelerators. Such optimizations would enable deployment on less than100 MB memory footprints and less than100 ms latency targets essential for practical large-scale PV monitoring networks.

Table 1 summarizes our model configuration and compares it with the traditional U-Net architecture using ResNet18 as one of the pretrained backbones (encoders) in the segmentation models of the Keras library that are trained on the ImageNet dataset. The compared model is the closest architecture to ours, and it has an approximate number of stages and total parameters, demonstrating that our model represents an enhancement over that model.

Additionally, the U-Net architecture is recognized for its precision in delineating object boundaries and its exceptional performance on small and costly-to-create datasets. Skip connections are critical components in this architecture, which combines the high-resolution, fine-grained spatial information from the encoder with the highly processed, semantically rich feature maps from the decoder. This allows the decoder to make precise, pixel-accurate localization decisions while having a deep understanding of the context. Collectively, these advantages render our proposed model more accurate, robust, and reliable for automated PV failure detection and localization.

**Table 1:** Summary of our proposed configuration.

Parameters	U-Net with ResNet18	Our hybrid model		
CNN kernel size (filters)	The first CNN 7 × 7 The rest CNNs 3 × 3	All 3 × 3		
No. of CNN residuals block in encoder	4 big blocks, each has 4 CNN and 2 residuals	5 cascaded, 3 parallel		
CNN layer depths Encoder	64–128-256–512	32–64-128–256-512, 3 × 200 = 600		
No. of down-samples	5 times	3 times		
No. of Transformer NN layers in encoder	--------	3 multilevel branches each has 3 cascaded layers, i.e. total = 9		
Transformers NN specs.	--------	**Branch1**	**Branch2**	**Branch3**
		*Input depth*: 256	128	512
		*h*: 6	4	2
		*MLP*: 128/64	64/32	32/16
No. of CNN residuals block in decoder	4 blocks, each has 2 CNN without residuals	3 blocks		
No. of up-samples	5 times	3 times		
CNN layer depths Decoder	512-256-128-64-32-16	256-128-64		
Total Parameters	14,344,630 ***Trainable***: 14,334,704	14,574,645 ***Trainable***: 14,562,405		

## Experiments and results

Experiments for the proposed model were conducted on the following hardware platform: NVIDIA GeForce RTX 3050 with 4 GB of GPU, Intel Core i7-11800 H at 2.30 GHz, and 16 GB of RAM. Windows 10 Pro, a 64-bit operating system, and the Keras framework with the TensorFlow 2.10.1 backend supported GPUs with CUDA toolkit 11.2 and CUDNN 8.1.0.

To assess the proposed strategy across several datasets, we compared our model’s performance with the following pretrained models: ResNet18 and VGG16 (Visual Geometry Group)^45^ as a backbone for the U-Net architecture to determine which one is best, as well as ResNet18 for the PSP-Net (Pyramid Scene Parsing Network)^46^, FPN (Feature Pyramid Networks)^47^, and LinkNet^48^ architectures. VGG16 is a strong baseline and simple architecture, which stacks multiple convolutional layers (with 3 × 3 filters) to increase depth, but it is significantly large, slow, and prone to the vanishing gradient problem in very deep networks; therefore, we limited its comparison to the U-Net architecture. PSP-Net architecture relies on building a pyramid of pooling at the end of the backbone to capture context at multiple scales, while LinkNet is designed to be efficient and fast because it uses additive skip connections (instead of the U-Net’s concatenation) between the encoder and decoder, which reduces computational cost. FPN is constructed as a top-down pathway with lateral connections to build a rich, multi-scale feature pyramid. The compared models and their descriptions are summarized in Table 2. Ultimately, recently published models utilizing identical datasets are introduced to demonstrate that our model surpasses their performance. For comparison results with published papers across all datasets, we did not use the same image size, batch size, or loss function. Some authors modify dataset images through preprocessing or by using different test sets, introducing variations that complicate direct comparison.

**Table 2:** Descriptions of compared models.

Backbone + Architecture model	Total Depth	Parameters (Millions)	Size (MB)	Computational Cost (GFLOPs)	Average Inference time (s)
*ResNet18 + U-Net*	~ 102	~ 14.3	~ 54.7	~ 4.4	~ 0.0843
*VGG16 + U-Net*	~ 65	~ 23.8	~ 90.6	~ 19.6	~ 0.0797
*ResNet18 + FPN*	~ 83	~ 13.8	~ 52.8	~ 19	~ 0.0845
*ResNet18 + LinkNet*	~ 117	~ 11.5	~ 44	~ 2.4	~ 0.0813
*ResNet18 + PSP-Net*	~ 61	~ 2	~ 8.4	~ 2.9	~ 0.0748
Proposed model	**~ 107**	**~ 14.5**	~ **55.6**	**~ 29.7**	~ **0.1125**

A loss function prevents the model from growing overconfident or depending too much on specific training samples, serving as a kind of regularization. The model learns to produce increasingly accurate predictions by reducing the difference between its outputs and the actual values through an iterative process of minimizing the loss. Weighted cross-entropy is a modified version of cross-entropy loss (traditional cross-entropy loss shown in Eq. 4) that assigns different weights to different classes, as in Eq. 5. It has widespread applications in semantic segmentation with imbalanced datasets. The Dice loss in Eq. 6 focuses on region-based optimization, as opposed to cross-entropy loss (which operates at the pixel level), which calculates how much the expected and ground truth masks overlap, making it well-suited for unbalanced datasets^49–51^.

4 $$\:{L}_{CE}\left(y,\widehat{y}\right)=-\left(\:y\mathrm{l}\mathrm{o}\mathrm{g}\left(\widehat{y}\right)+\left(1-y\right)\mathrm{l}\mathrm{o}\mathrm{g}\left(1-\widehat{y}\right)\right)=-\frac{1}{N}\sum\:_{i=1}^{N}\sum\:_{j=1}^{C}\left({y}_{i,j}\mathrm{log}\left({\widehat{y}}_{i,j}\right)\right)$$

where *y* and *ŷ* are the true and predicted values of a sample, respectively, *i* denotes a sample image of total *N*, and *j* denotes a class of total *C* for multiclass problems.

$$\:{L}_{WCE}\left(y,\widehat{y}\right)=-\left(\beta\:*\:y\:\mathrm{l}\mathrm{o}\mathrm{g}\left(\widehat{y}\right)+\left(1-\beta\:\right)\left(1-y\right)\mathrm{log}\left(1-\widehat{y}\right)\right)\:$$

5 $$\:=-\frac{1}{N}\sum\:_{i=1}^{N}\sum\:_{j=1}^{C}\left({w}_{j}\:{y}_{i,j}\mathrm{log}\left({\widehat{y}}_{i,j}\right)\right)$$

where *β* ε [0, 1] is the factor value that penalizes for a specific class, and *w* is the weight assigned to a class *j*.

6 $$\:DL\left(y,\widehat{y}\right)=1-Dice=1-\frac{2\left[y\cap\:\widehat{y}\right]+\epsilon\:}{y\cup\:\widehat{y}+\epsilon\:}=1\:-\frac{2y\widehat{y}+\:1\:}{y\:+\widehat{y}\:+\:1}\:\:$$

All dataset images are resized to 160 × 160 × 3 pixels, and with batch sizes of 2, we start training our model using a 0.0001 learning rate and the optimizer of Adam, which is able to modify the learning rate for every single parameter. The size of images and batches are chosen according to the hardware limitations.

Evaluation metrics are employed to evaluate the suggested methodology’s performance. The predictions of testing the suggested model are assessed using measures such as global pixelwise accuracy, mIoU, precision, recall^52^, and F1-score (weighted and unweighted). All of these pertain to true positives (TP), false positives (FP), true negatives (TN), and false negatives (FN). A TP indicates that the model accurately classifies a certain PV fault class in all pixels, whereas a TN indicates that the model correctly classifies the opposite PV faults (or classes) in pixels. Conversely, FP indicates that the model classifies the class wrongly, while FN denotes the inaccurate classification of the opposite classes.

In semantic segmentation (pixelwise classification), the accuracy statistic indicates how often a model is correct overall (predicted true pixels) in Eq. 7. The precision metric indicates how often a model properly predicts a target class in Eq. 8, and the recall metric evaluates the percentage of correctly real positive cases that a model properly detects in Eq. 9. Equation 10 has the model performance parameter of F1-score (Dice coefficient), which integrates precision and recall, evaluates the correctness of both positive and negative pixel classifications in the segmentation outcomes (calculates the images’ similarity). The weighted F1-score (*wDice/wF1*) was employed to assess the models, accounting for class imbalance in the dataset, which computes the F1-score for each class and subsequently averages them using weights proportionate to the number of true cases for each class. The Intersection over Union (IoU) (Jaccard index) in Eq. 11 quantifies the overlap between expected and actual segmentation masks, assessing the precision of fault localization. We have used three types of values: the mean/unweighted (*mAP*) or macro values, which sum the individual values for all samples in each class and calculate their average without accounting for unbalanced classes; the weighted values (*wIoU*), which incorporate the ratio of the number of pixels in each class as a weight in calculations; and the global values (*gF1*) or micro, which compute totals across all classes at once.

7 $$\:Global\:Accuracy\:\left(Pixelwise\right)=\frac{TP+TN}{TP+TN+FP+FN}$$

8 $$\:Precision=\frac{TP}{TP+FP}$$

9 $$\:Recall=\frac{TP}{TP+FN}$$

10 $$\:F1\:score=Dice=\frac{2\:Precision\:*\:Recall}{Precision+\:Recall}=\frac{2\:TP}{2\:TP+FP+FN}$$

11 $$\:IoU=\frac{y\cap\:\widehat{y}}{y\cup\:\widehat{y}}=\frac{TP}{TP+FP+FN}$$

### Thermal dataset results

The thermal dataset that indicates the faulty cells in the PV system has a highly imbalanced pixel distribution, as the faulty class areas (faulty PV cells) are fewer than the non-faulty ones due to the relatively small area occupied by faulty regions. In that case, the model training is significantly challenged, resulting in biased predictions towards the majority class towards the non-faulty class. Our model, along with all the other models being compared, is trained, validated, and tested using a combined loss function of weighted categorical cross-entropy and Dice loss. This strategy enhances model performance by combining the two losses, as shown in Eq. 12^50^. Also, combining unweighted cross-entropy loss with dice loss when training the second dataset enhances the results (replacing *L*_*WCE*_ with *L*_*CE*_ in Eq. 12).

12 $$\:CL\left(y,\widehat{y}\right)={\alpha\:L}_{WCE}\left(y,\widehat{y}\right)+\left(1-\alpha\:\right)DL\left(y,\widehat{y}\right)$$

where *α* controls the amount of contribution of both losses.

All the trained hyperparameters of the model according to the four investigated datasets are illustrated in Table 3.

**Table 3:** Detailed hyperparameter settings.

Hyperparameter Item	Value			
Input shape Images	160 × 160 × 3			
Output activation function	SoftMax			
Dense activation function	ReLU in CNN, and GELU in Transformer			
Parameters of Normalization	Epsilon = 10^− 6^ in Transformer			
Batch size	2			
Optimizer	Adam			
Learning rate	10^− 4^			
Cross Validation Split	Thermal dataset	PVEL dataset	UCF-EL	Benchmark EL
	20% for validation and 10% for test	30%	10% for validation and 10% for test	Customized according to data collectors
Epochs	More than 100	More than 60	More than 50	More than 100
Loss Function	Combined loss *CL = α L*_*WCE*_ *+(1-α) DL* at α = 0.6, and customized w= [1,10]	Combined loss *CL = α L*_*CE*_ *+(1-α) DL* at α = 0.6, and *L*_*CE*_ has label smoothing = 10^− 4^	*L*_*WCE*_ with customized weights = [1, 2, 3,10,4]	*L*_*WCE*_ with equal weights of 1’s

**Fig. 7:**
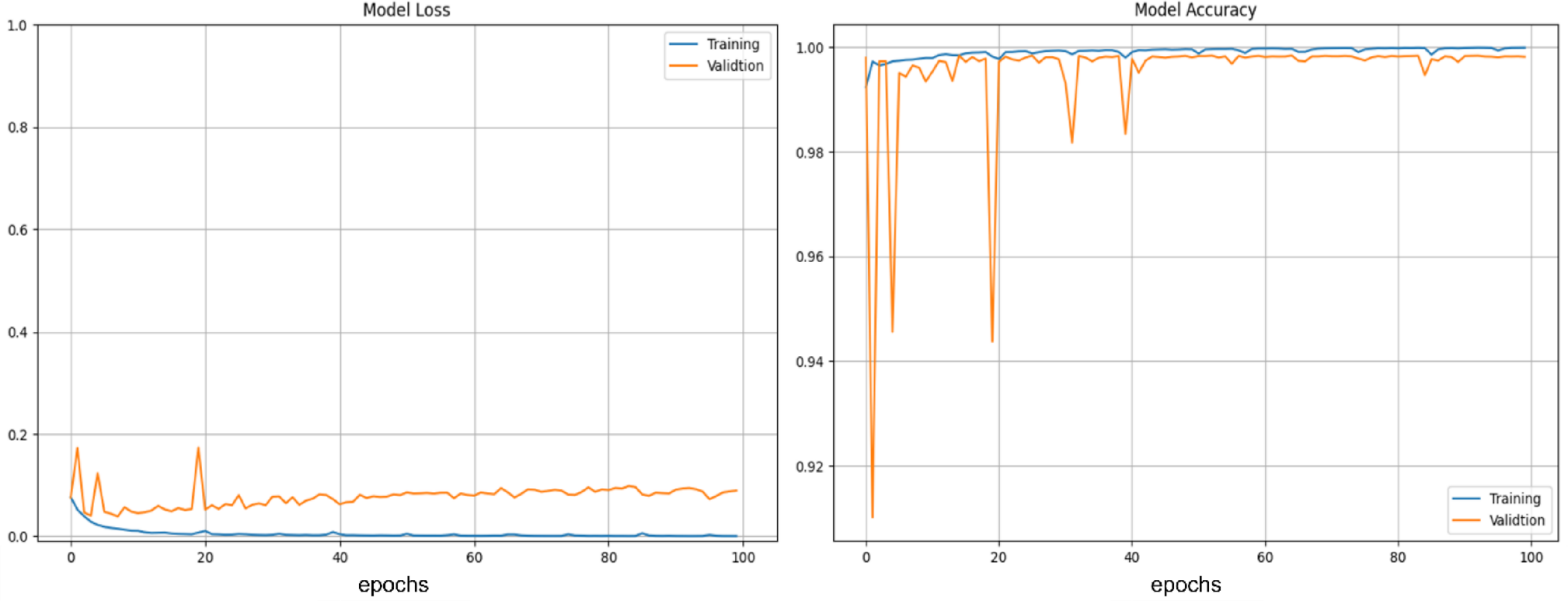
Training and validation performance of thermal dataset.

**Fig. 8:**
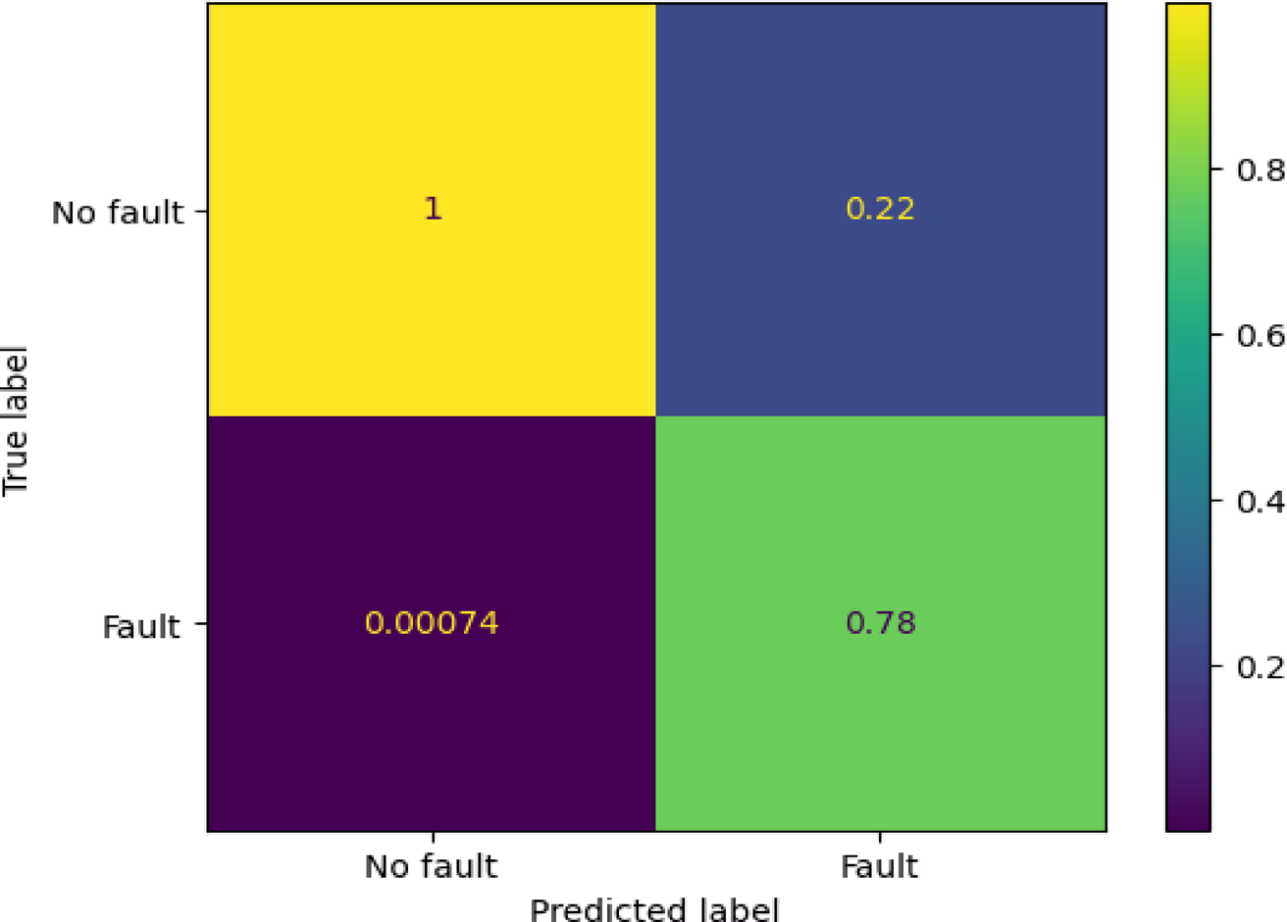
Confusion matrix for thermal dataset.

Training and evaluation procedures were implemented independently for each of the compared models to establish comprehensive fault localization models employing diverse AI approaches. This validation strategy confirms the efficacy of the proposed model and enables comparative performance analysis across all models. The performance of training and validation of our model for the first thermal imaging dataset is shown in Fig. 7, which indicates that the accuracy improves and stabilizes around epoch 50. A testing phase was conducted to assess the proposed methodology on unseen images from the dataset, as summarized in Table 4 and visualized in the confusion matrix (Fig. 8). We also compared it with other models, including published papers using the same dataset. All the results are combined in Table 5.

**Table 4:** Testing results per class for the thermal dataset.

Class	Precision	Recall	F1-score	No. of pixels
Background	0.999258	0.999647	0.999452	2,580,536
Faulty cells	0.775425	0.621840	0.690192	5064

**Table 5:** Metrics for evaluating our model versus other models for the thermal dataset.

Model	Accuracy	mAP	mRecall	mDice	mIoU
ResNet18 + U-Net	0.9987	0.8708	0.7718	0.8137	0.7282
VGG16 + U-Net	0.9982	0.7669	0.8328	0.7962	0.7100
ResNet18 + FPN	0.9988	0.8590	0.7948	0.8235	0.7388
ResNet18 + LinkNet	0.9981	0.7566	0.7856	0.7703	0.6847
ResNet18 + PSP-Net	0.9981	0.4995	0.4995	0.4995	0.4990
**Proposed model**	**0.9989**	**0.8873**	**0.8107**	**0.8448**	**0.7629**
EfficientNet + U-Net^36^	--------	--------	--------	0.8410	0.7410
ASPP + Attention + ResNet + U-Net^18^	0.9970	**0.9110**	**0.8400**	**0.8680**	**0.7900**

The results indicate that our proposed model achieves the highest accuracy of 99.89% among all the compared models, demonstrating its efficacy in accurately localizing faulty cells with a mean precision (mAP) of 88.73%, a mean dice (mDice) of 84.48% and a mean recall (mRecall) of 81.07%. While traditional ResU-Net or FPN models are the second-best results, PSP-Net exhibited training difficulties, resulting in the poorest performance.

Our model demonstrated superior performance compared to other models, although when comparing our model with the model in^18^, the comparison may not be equitable because the authors applied image enhancements (Contrast Limited Adaptive Histogram Equalization) and data augmentation that resulted in increasing performance of mIoU and mAP by about 3% more than ours, suggesting that thermal images require additional preprocessing due to the huge misbalancing of classes. The test set contains over 2.5 million pixels, but only about 5 thousand of these are faulty, representing approximately 0.2% of the total, which highlights the unbalancing problem present in the rest of the dataset. Also, the quality of the thermal image needs to increase defect visibility without creating noticeable artifacts, as some regions exhibit less variation^18^. Dataset enhancements would be considered in the future approach of our fault detection and localization system.

**Fig. 9:**
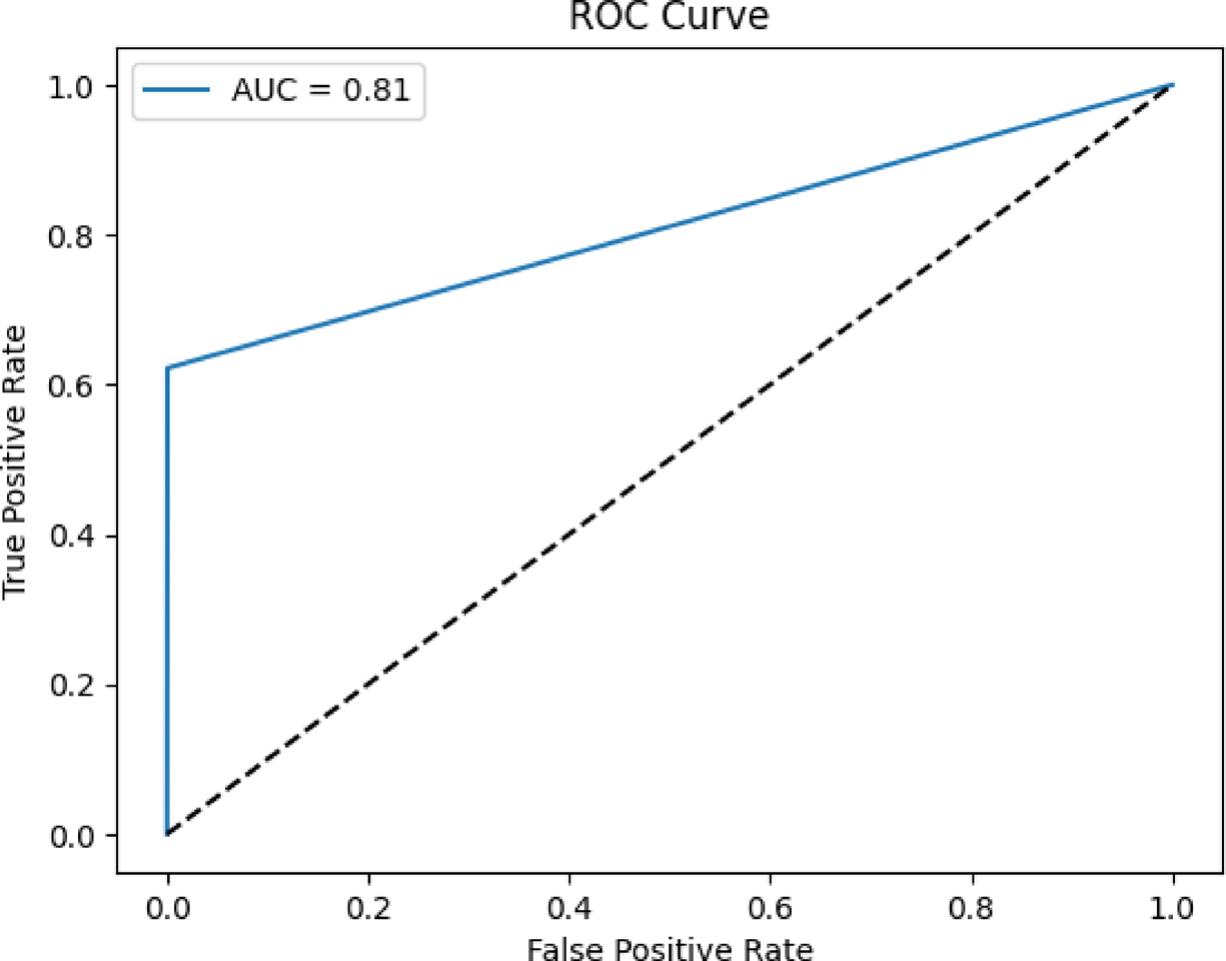
ROC and AUC curves for the thermal dataset.

The performance for segmentation is also measured using the Receiver Operating Characteristic Curve (ROC) and area under the ROC curve (AUC) in Fig. 9. An AUC of 81% indicates satisfactory discrimination capability and separability.

**Fig. 10:**
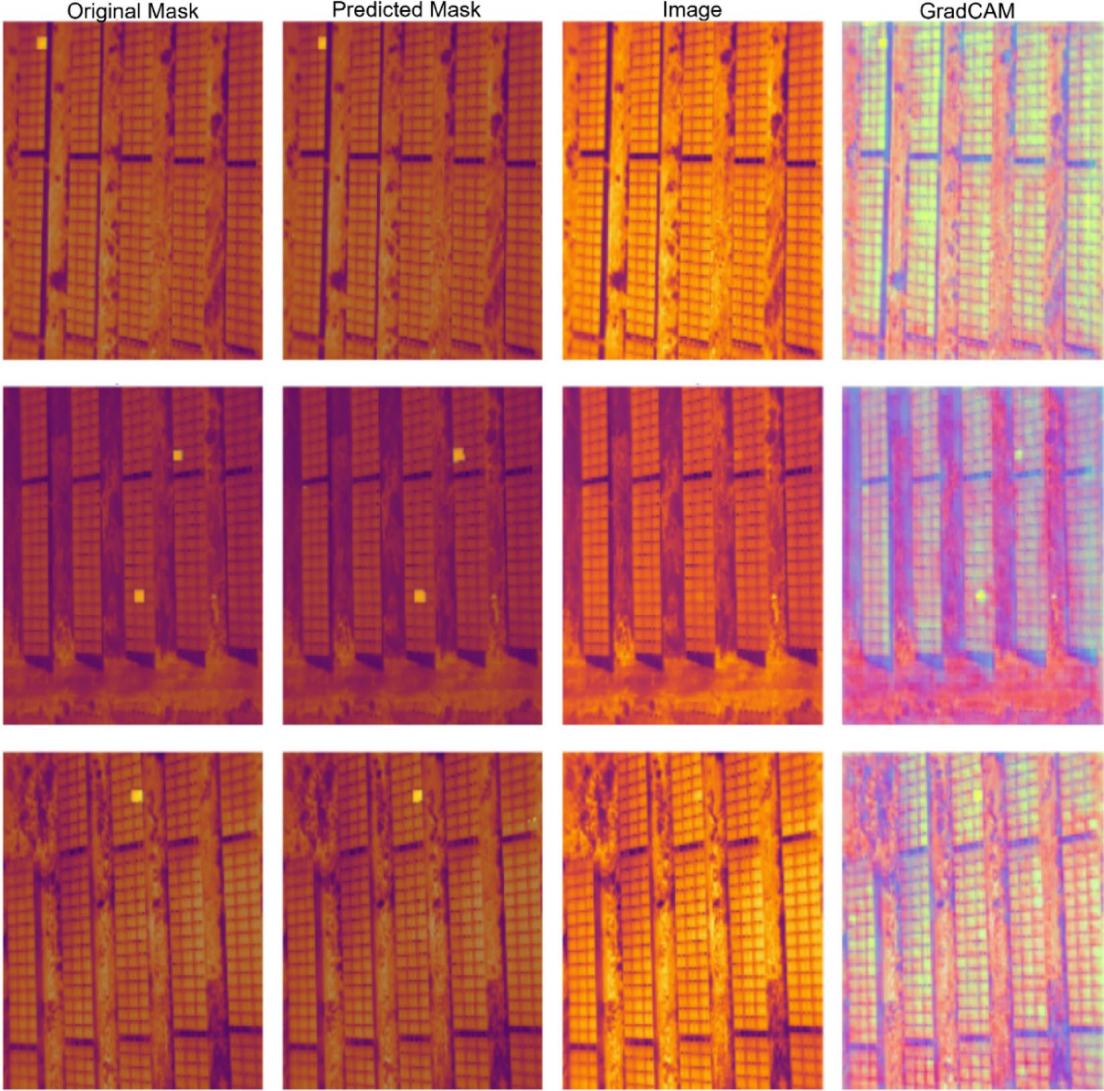
Visualizing some predicted images and their Grad-CAM of the tested images for the thermal dataset.

Figure 10 displays the predicted masked images and their Gradient-Class Activation Map (Grad-CAM) heatmaps for a few tested images. Heatmaps are superimposed with the original images to illustrate the dependencies from the segmented classes that allow us to gauge how sensitive our model is to the input images. The figure shows a perfect predicted segmentation, which ensures our system’s ability to aid in fault localization of cells.

### PVEL-S dataset results

**Fig. 11:**
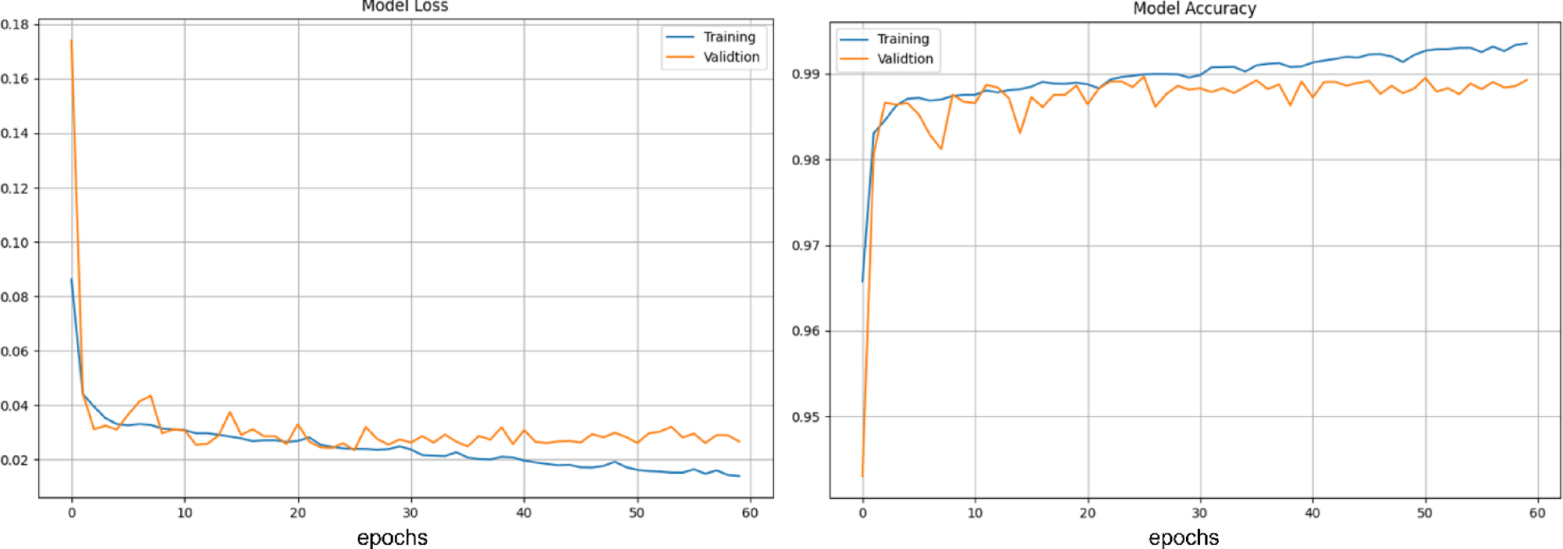
Training and validation performance of PVEL-S dataset.

The PVEL-S dataset, which identifies defective regions in the PV cell, was investigated and also exhibits class imbalance, but with a moderate ratio; we employed a combined loss function to address this challenge. Model accuracy decreased when combining Dice loss with weighted cross entropy loss, so we combined the unweighted categorical cross entropy at label smoothing of 0.0001 with the Dice loss, which improved model performance without needing any data augmentation, as illustrated in Fig. 11. The effect of label smoothing improves the accuracy by more than 1% and increases model robustness against label noise, also preventing the model from becoming overconfident. For future work, we would be investigating an adaptive soft labelling method, which might better handle fine-grained defect boundaries for other datasets.

**Fig. 12:**
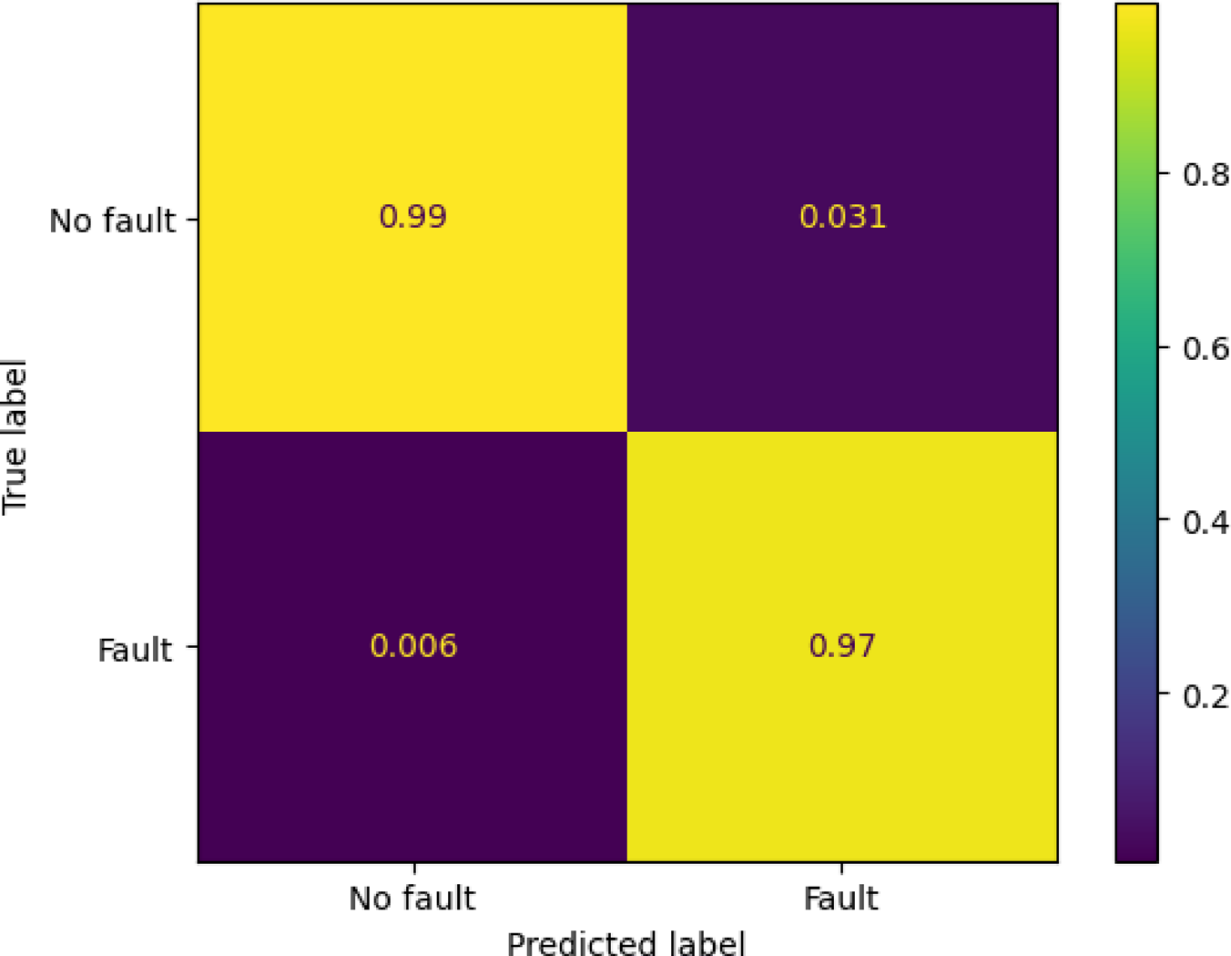
Confusion matrix for PVEL-S dataset.

To evaluate our model for the second dataset, testing was performed to validate model training effectiveness, as illustrated in Fig. 12; Table 6, which show that defective area segmentation achieved satisfactory results with 96.88% precision. Furthermore, the comparison results demonstrate our model’s superiority over all benchmark models, as shown in Table 7.

**Table 6:** Testing results per class for the PVEL-S dataset.

Class	Precision	Recall	F1-score	No. of pixels
Non-Defected areas	0.994016	0.994137	0.994076	7,756,995
Defected areas	0.968807	0.968182	0.968495	1,459,005

**Fig. 13:**
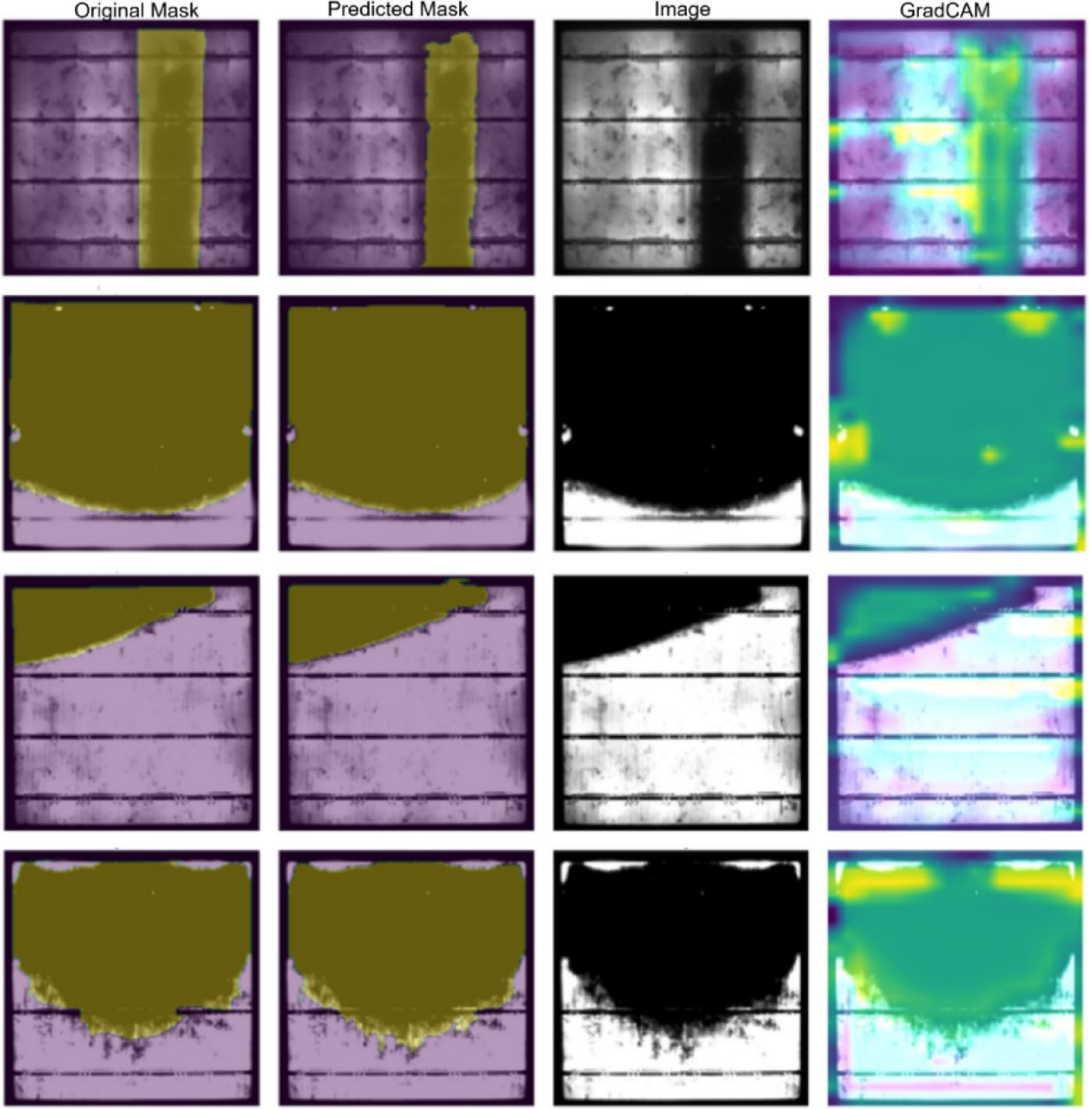
Visualizing some predicted images and their Grad-CAM for the encoder for the PVEL-S dataset.

With an accuracy of 99% and an mDice of 98.13%, our model has the best performance ever, even compared to the published paper in^19^ that used data augmentation. The second-best performance is the FPN and LinkNet models. Although our model mean recall (mRecall) does not have the best score, the weighted and global recall of 99% is the highest value of the rest of the models. Heatmaps for the encoder layers in Fig. 13, which show the important defective areas, have been highlighted perfectly. This confirms that the transformer layers added more global details to the encoder, resulting in impressive performance against all the compared models.

**Table 7:** Metrics for evaluating our model versus other models for the PVEL-S dataset.

Model	Accuracy	mAP	mRecall	mDice	mIoU
ResNet18 + U-Net	0.9888	0.9759	0.9825	0.9791	0.9595
VGG16 + U-Net	0.9881	0.9728	**0.9830**	0.9778	0.9570
ResNet18 + FPN	0.9888	0.9764	0.9817	0.9790	0.9593
ResNet18 + LinkNet	0.9888	0.9763	0.9820	0.9791	0.9595
ResNet18 + PSP-Net	0.9886	0.9763	0.9812	0.9787	0.9587
**Proposed model**	**0.9900**	**0.9814**	0.9812	**0.9813**	**0.9636**
K-Net+ CBAM + SE + ARM+ ASPP^19^	0.9795	0.9801	--------	0.9798	0.9607

**Fig. 14:**
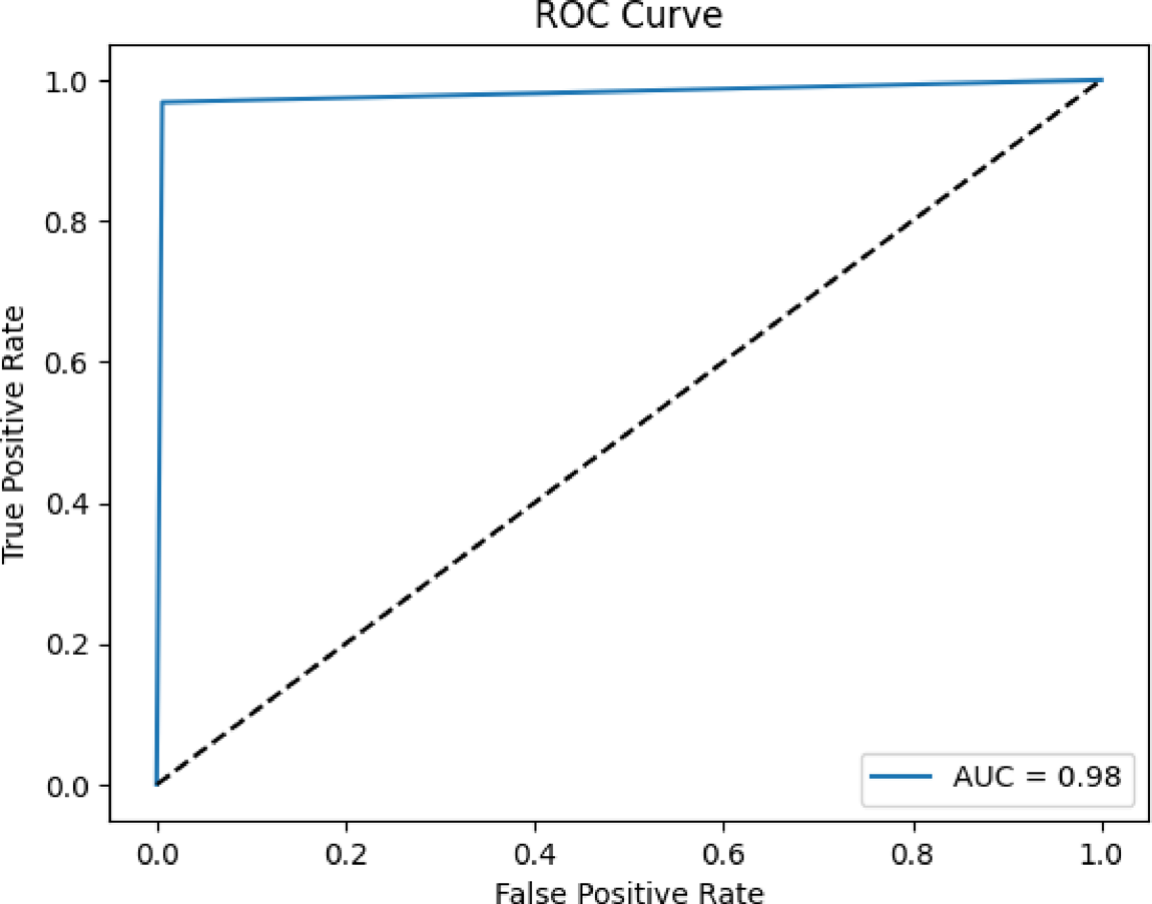
ROC and AUC curves for PVEL-S dataset.

With a fabulous discrimination AUC of 98% in Fig. 14, and observing the predicted image results in Fig. 13, we find that the proposed model has localized the PV defective regions on cells for the PVEL-S dataset with excellent performance and minor errors.

### UCF-EL dataset results

**Fig. 15:**
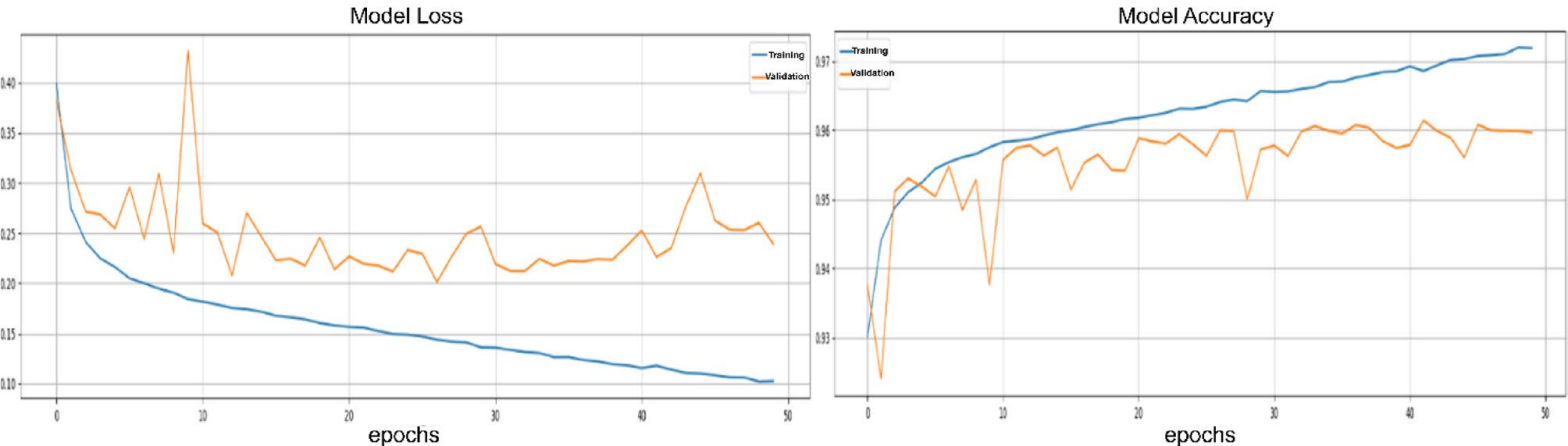
Training and validation performance of UCF-EL dataset.

The UCF-EL dataset comprises five PV fault classes with an unbalanced number of pixels; thus, a weighted cross-entropy loss function with customized weights was employed to address the class imbalance and enhance model performance, as illustrated in Fig. 15. We observed that the weighted loss function improves the overall results of our model but had a more pronounced impact on the benchmark models. This can be attributed to the multi-scale architecture of our Transformer layers, which results in more enhancement using the unweighted loss function, as in the second dataset results.

**Fig. 16:**
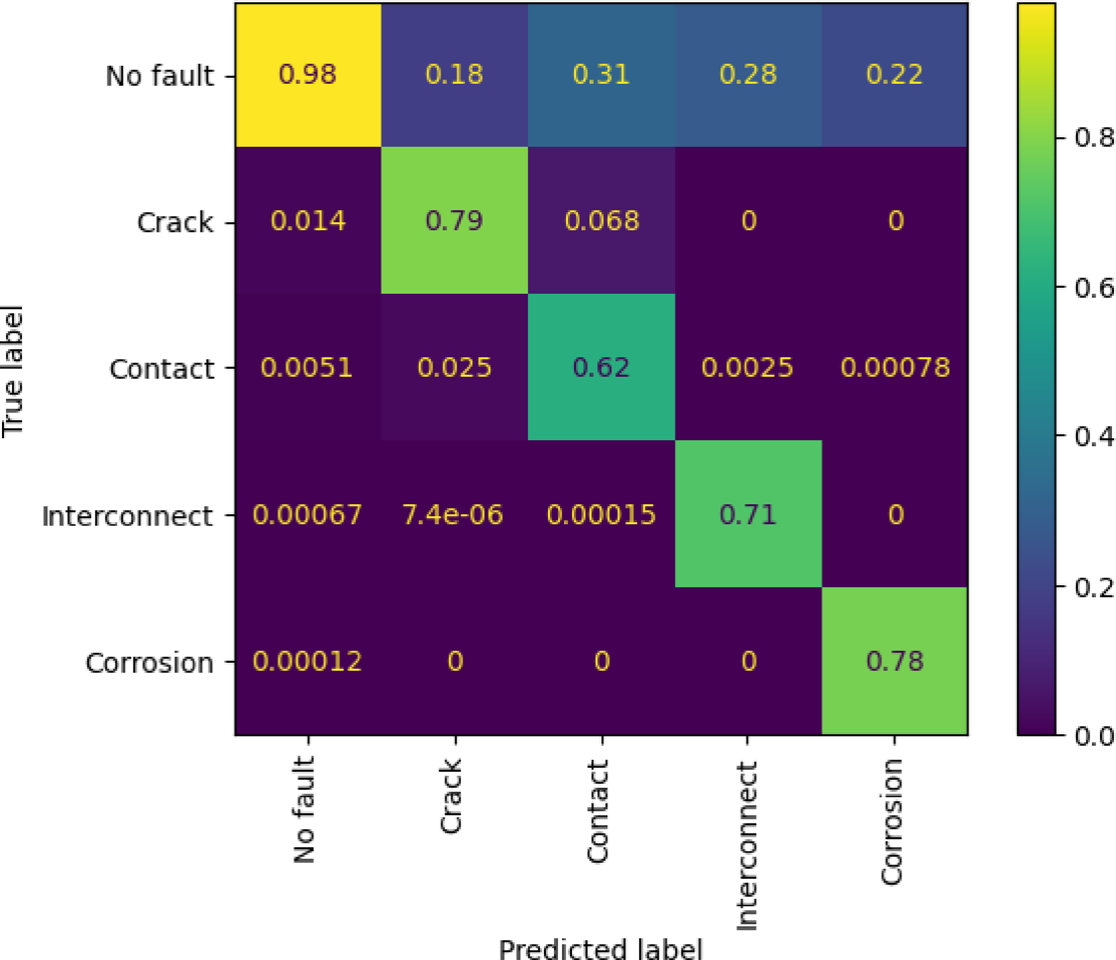
Confusion matrix for UCF-EL dataset.

Testing our model results can be found in Table 8; Fig. 16, which give the details of the segmentation accuracy, showing that the cracks and corrosion are the most efficient PV failure localization results.

**Table 8:** Testing results per class for the UCF-EL dataset.

Class	Precision	Recall	F1-score	No. of pixels
No fault	0.9801	0.9823	0.9812	26,964,649
Crack	0.7931	0.7716	0.7822	1,797,898
Contact	0.6168	0.6223	0.6195	482,753
Interconnect	0.7143	0.1383	0.2317	20,989
Corrosion	0.7776	0.8411	0.8081	20,111

Comparative results with state-of-the-art models are presented in Table 9. The results show that our model’s performance has improved, especially compared to published papers using the same dataset, with an accuracy of 96.27% and a mean precision of 77.64%, achieving the highest precision among all models. However, the precision improvement came at the expense of a reduced Dice score.

The VGG-U-Net and FPN models also demonstrated competitive performance for mean recall, dice and IoU scores. Future work will explore alternative architectures or backbone encoders that can be improved by incorporating transformer neural networks to enhance dice performance in model segmentation for EL images containing minor PV faults, which are challenging to detect even for experienced human observers or through image enhancement prior to training.

**Table 9:** Metrics for evaluating our model versus other models for the UCF-EL dataset.

Model	Accuracy	wF1	mAP	mRecall	mDice	mIoU
ResNet18 + U-Net	0.9617	0.9611	0.7220	0.6767	0.6900	0.5682
VGG16 + U-Net	0.9619	0.9623	0.6839	**0.7203**	0.6826	0.5624
ResNet18 + FPN	0.9614	0.9608	0.7051	0.6957	**0.6954**	0.5726
ResNet18 + LinkNet	0.9585	0.9581	0.7481	0.6115	0.6479	0.5291
ResNet18 + PSP-Net	0.9552	0.9542	0.6903	0.6176	0.6283	0.5164
**Proposed model**	**0.9627**	**0.9624**	**0.7764**	0.6711	0.6845	**0.5730**
ResNet50 + Deeplabv3^20^	0.9540	0.9500	--------	--------	0.6900	0.5730
SegFormer^11^	0.9620	0.9620	--------	--------	**0.8200**	0.5650

**Fig. 17:**
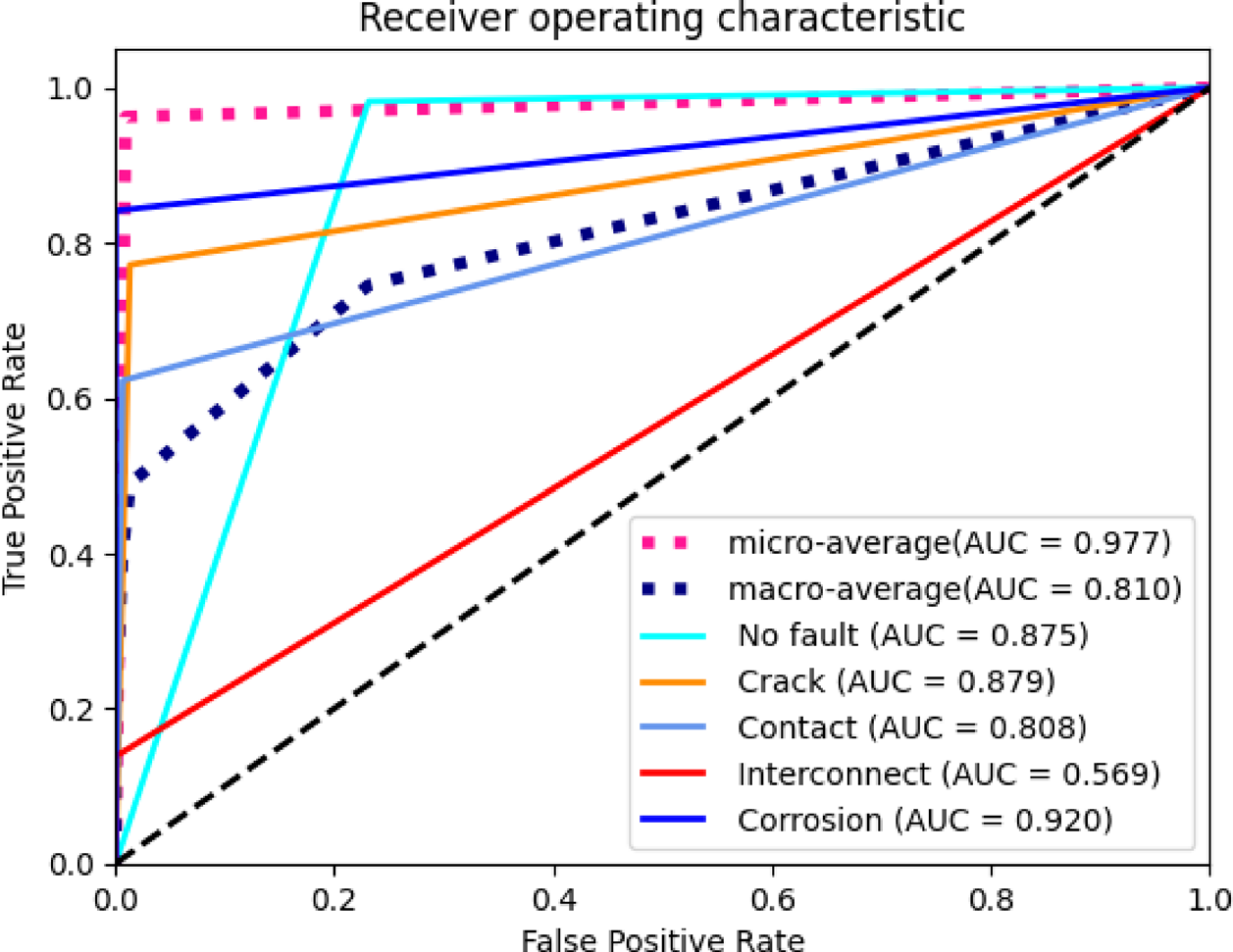
ROC and AUC curves for the UCF-EL dataset.

**Fig. 18:**
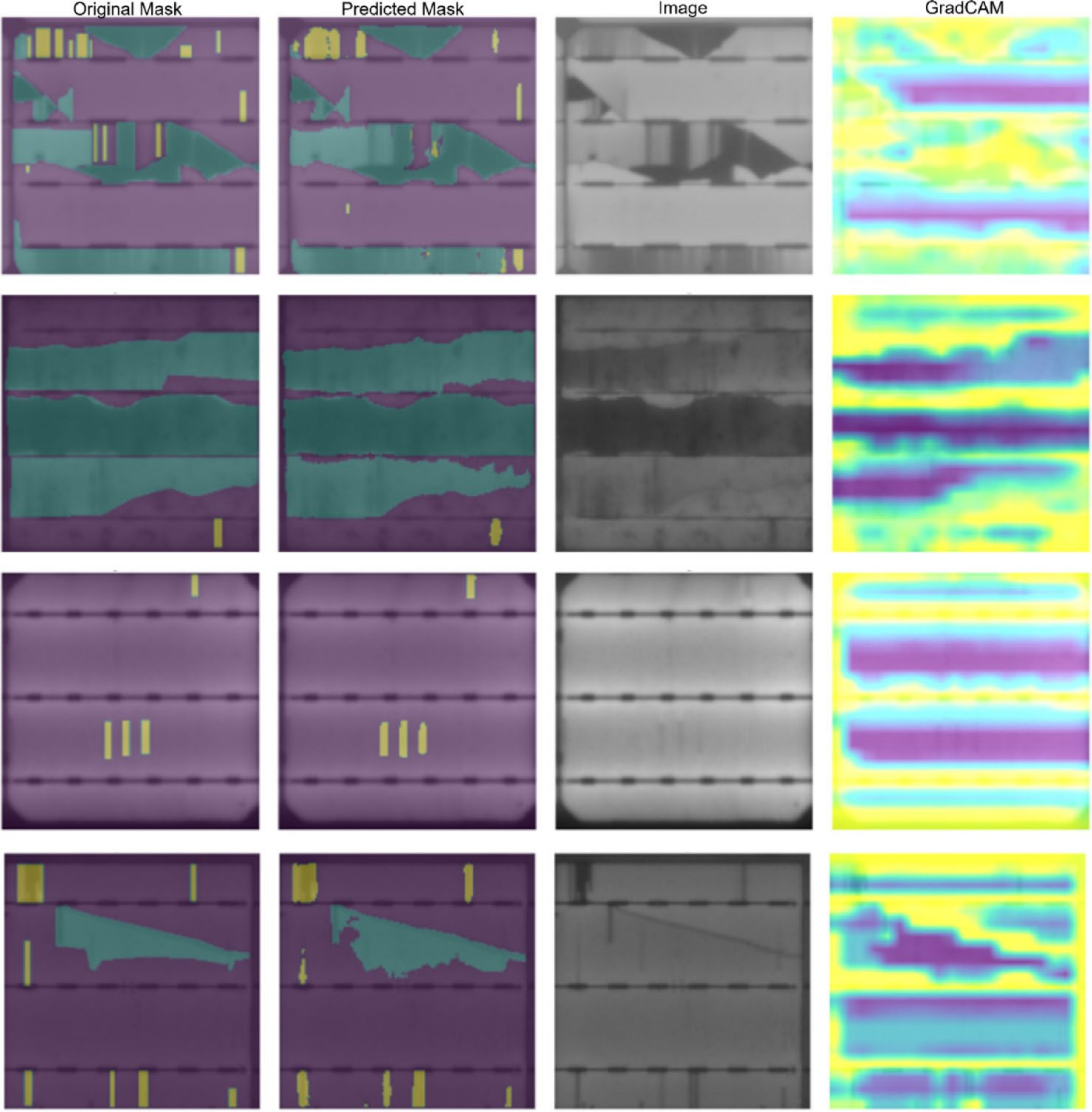
Visualizing some predicted images and their Grad-CAM for the encoder for the UCF-EL dataset.

The mean and global AUC, and also their values per class, are shown in Fig. 17, which confirms the model’s good overall discrimination ability of 97.7% for classes and excellent performance on most of them. The predicted image samples and their Grad-CAM can be found in Fig. 18, which shows a perfect multiclass segmentation demonstrating that our model is perfect for localizing almost all PV fault classes.

### Benchmark EL dataset results

**Fig. 19:**
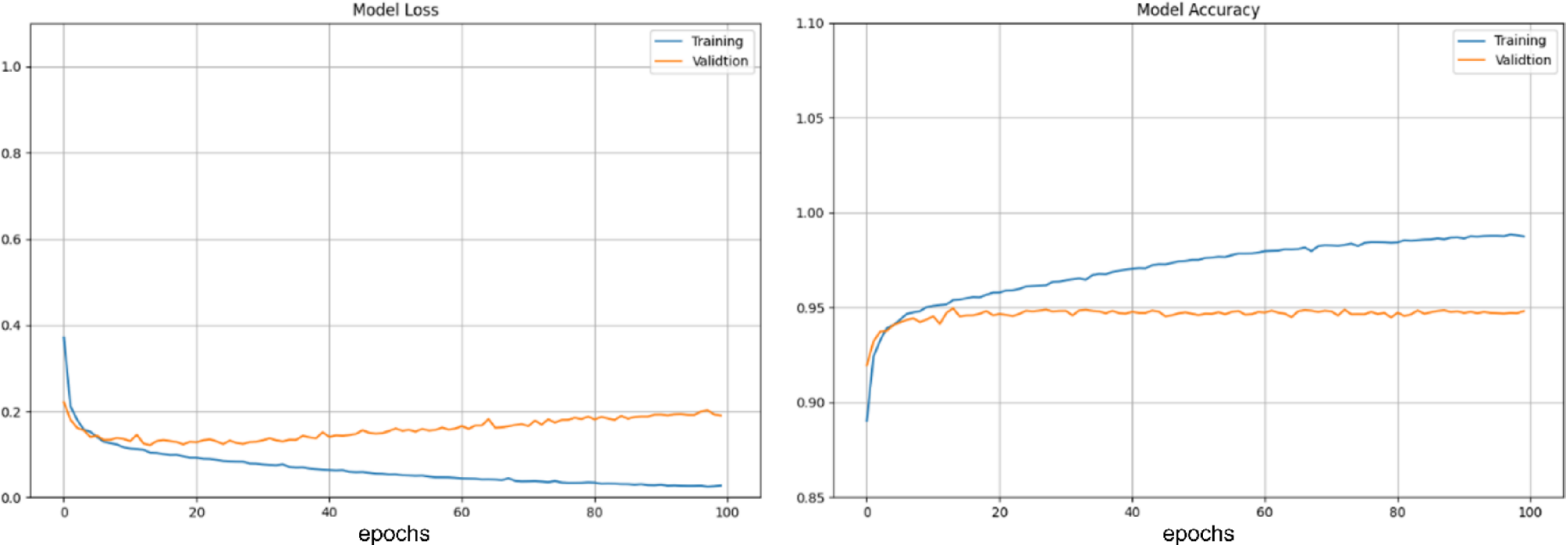
Training and validation performance of Benchmark EL dataset.

The benchmark dataset also has unequal numbers of classes, and it requires a weighted entropy loss function with equal weights of 1, as the model’s precision is affected by customized weights. The model training performance is illustrated in Fig. 19.

**Fig. 20:**
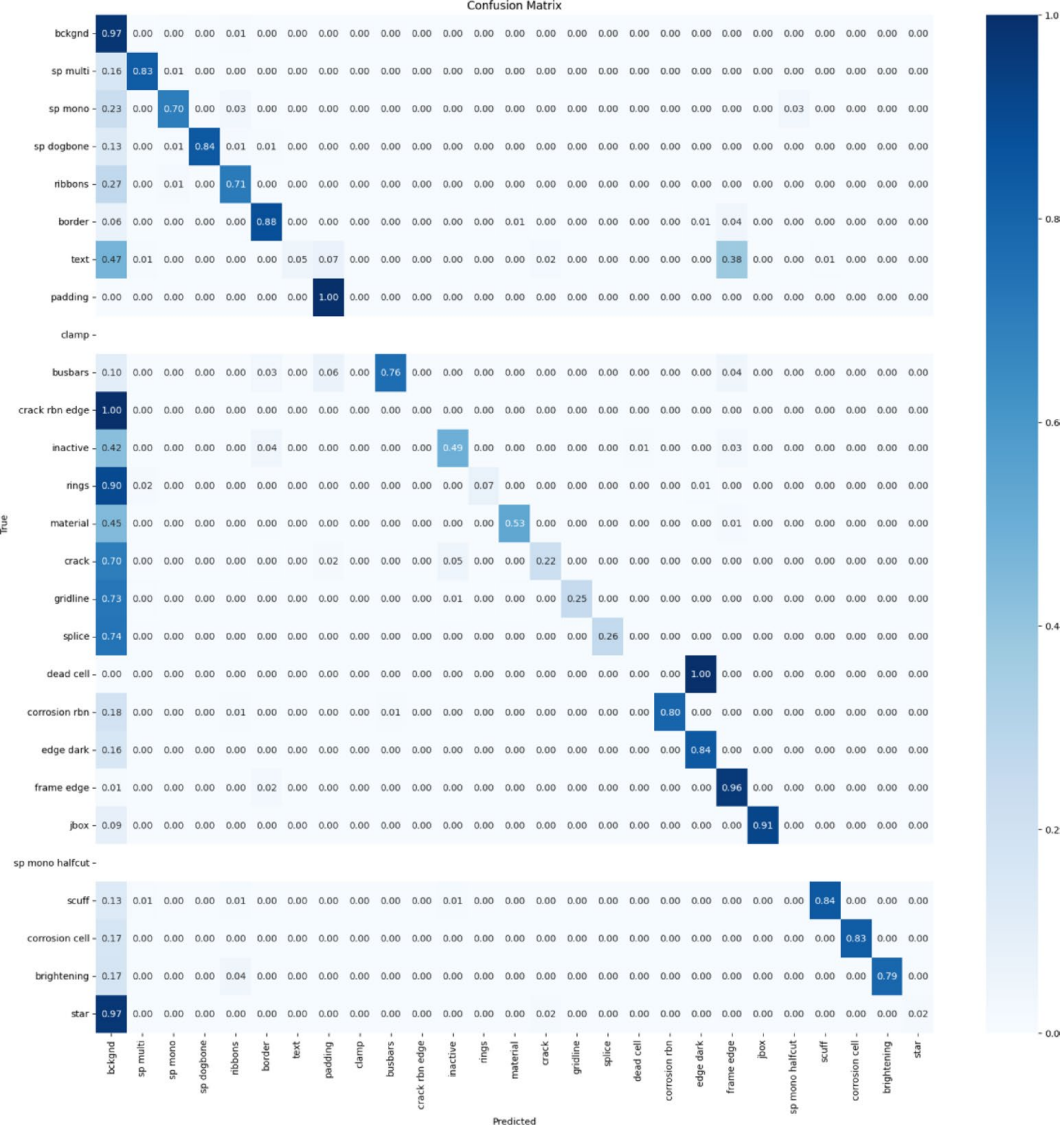
Confusion matrix for Benchmark EL dataset.

Model testing results are shown in Fig. 20; Table 10. The dataset unbalancing here is a big issue due to the number of classes and their various sizes. The results of only 25 classes have the highest defect segmentation of scuff and corrosion ribbon and the highest cell features of padding and junction box.

**Table 10:** Testing results per class for the Benchmark EL dataset.

Class	Precision	Recall	F1-score	No. of pixels
bckgnd	0.9626	0.9729	0.9677	1,292,953
sp multi	0.8477	0.8307	0.8391	29,439
sp mono	0.7702	0.7006	0.7338	35,193
sp dogbone	0.8948	0.8417	0.8675	2,679
ribbons	0.7595	0.7127	0.7354	60,520
border	0.9108	0.8817	0.8961	29,369
text	0.3600	0.0504	0.0885	357
padding	0.9982	0.9972	0.9977	343,073
busbars	0.8263	0.7607	0.7921	1,876
crack rbn edge	0	0	0	6
inactive	0.7460	0.4942	0.5945	6,016
rings	0.6232	0.0733	0.1311	587
material	0.6508	0.5339	0.5866	7,056
crack	0.4643	0.2189	0.2975	3,477
gridline	0.5608	0.2522	0.3479	8,188
splice	0.6849	0.2586	0.3755	580
dead cell	0	0	0	2
corrosion rbn	0.8492	0.7958	0.8216	1,479
edge dark	0.4134	0.8406	0.5542	1,951
frame edge	0.8145	0.9611	0.8817	11,980
jbox	0.9521	0.9145	0.9329	152
scuff	0.8201	0.8399	0.8299	912
corrosion cell	0.6977	0.8315	0.7587	2,670
brightening	0.7399	0.7873	0.7629	2,619
star	0.3333	0.0152	0.0290	66

**Fig. 21:**
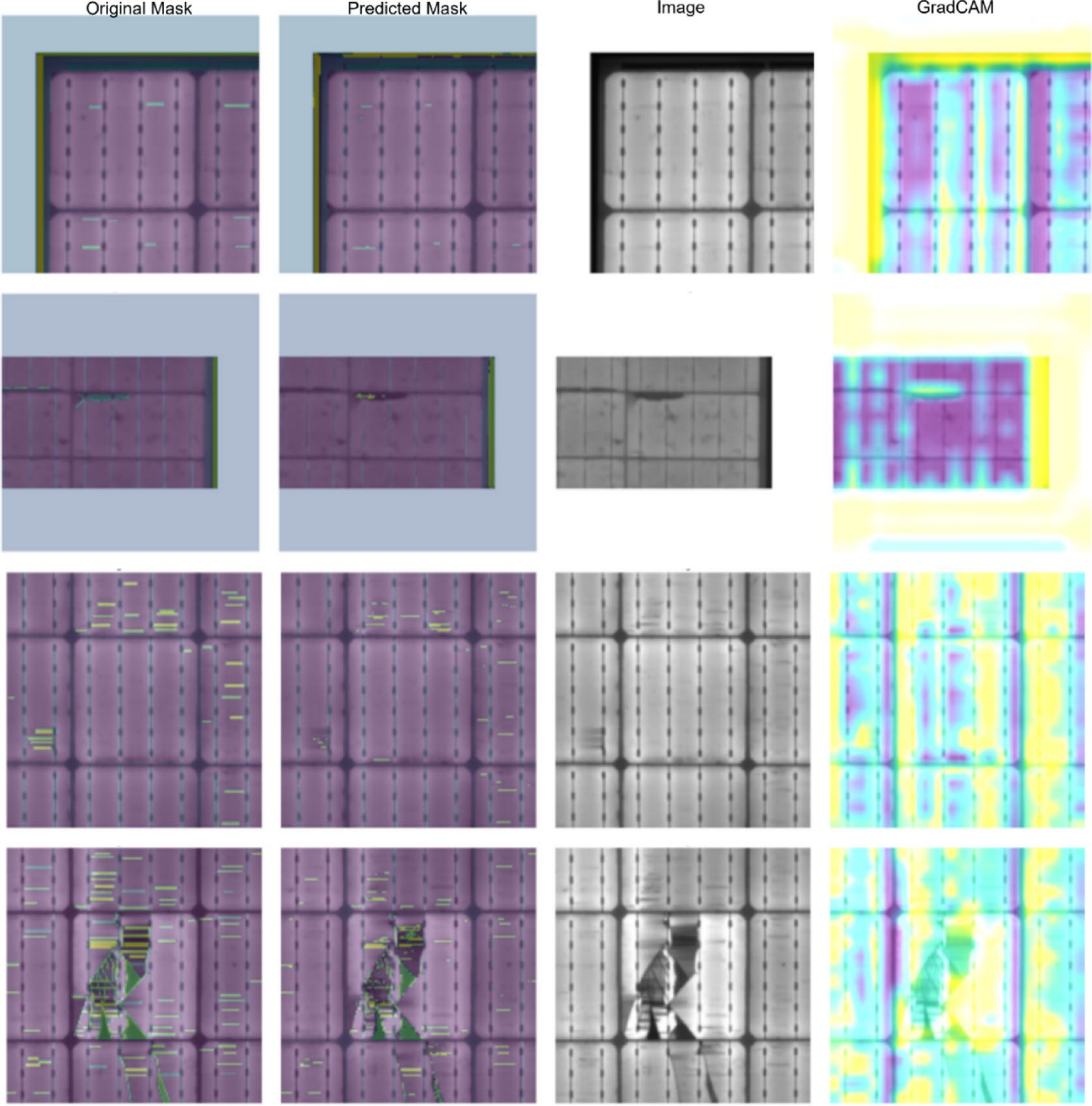
Visualizing some predicted images and their Grad-CAM for the encoder for the Benchmark EL dataset.

**Fig. 22:**
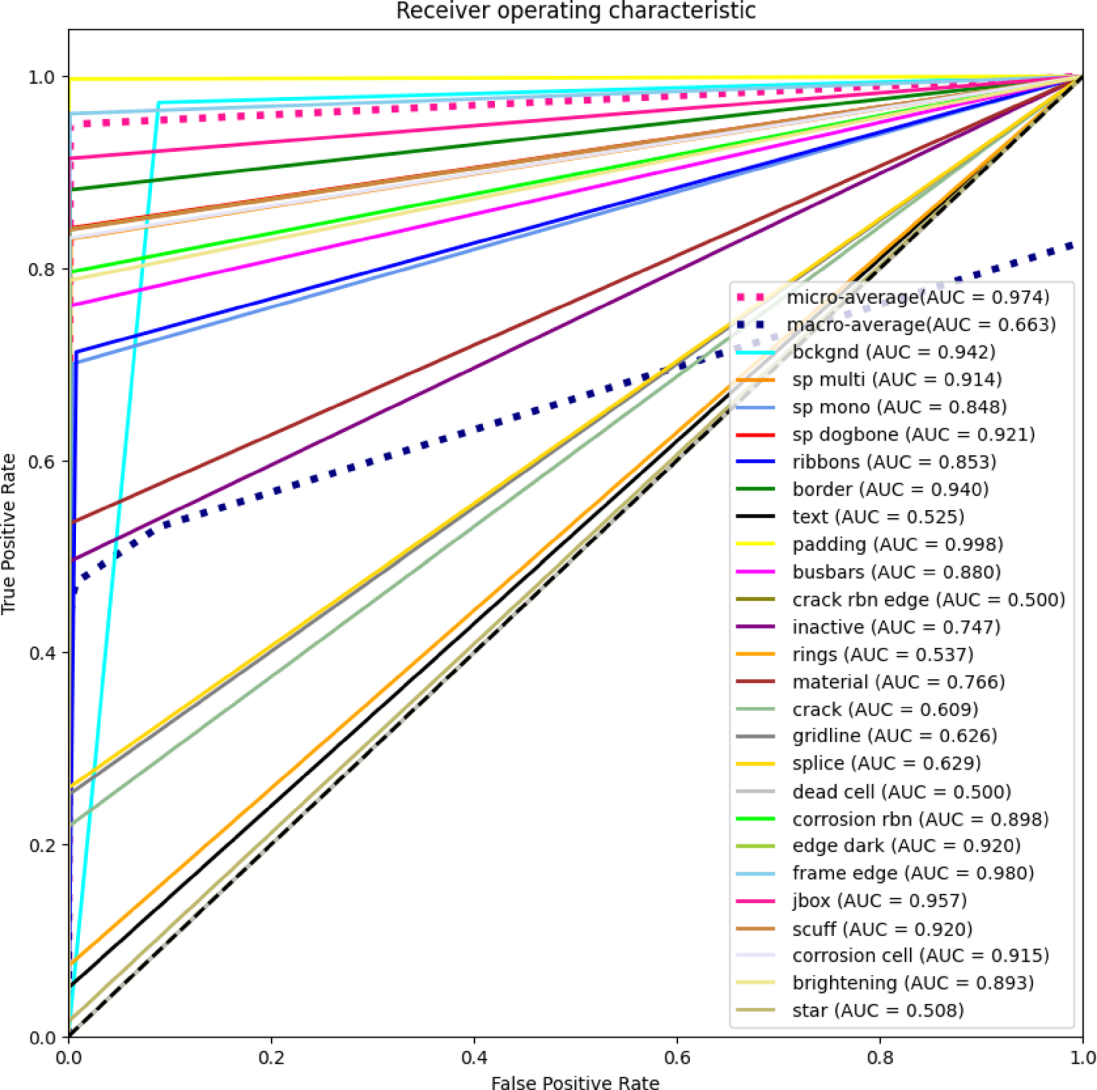
ROC and AUC curves for the Benchmark EL dataset.

According to Table 11, which compares our model performance with other models, with 95% accuracy and 66.7% mean precision, ours has the best results among the other comparative models and a close performance to the published one in^22^. To show the results as the predicted images and their heatmaps in Fig. 21 and, moreover, in Fig. 22, which shows the global AUC of 97.4%.

**Table 11:** Metrics for evaluating our model versus other models for the Benchmark EL dataset.

Model	Acc.	wIoU	gP	gF1/wF1	gIoU	gRec.	mAP	mRecall	mDice	mIoU
Res-UNet	0.946	0.905	0.946	0.946/0.944	0.897	0.946	0.634	0.503	0.514	0.416
VGG-UNet	0.947	0.908	0.947	0.947/0.946	0.899	0.947	0.602	0.549	0.552	0.459
FPN	0.948	0.909	0.948	0.948/0.946	0.901	0.948	0.682	0.533	0.559	0.458
LinkNet	0.943	0.900	0.943	0.943/0.940	0.892	0.943	0.569	0.488	0.504	0.407
PSP-Net	0.905	0.830	0.906	0.905/0.884	0.826	0.905	0.538	0.344	0.377	0.293
**Proposed**	**0.950**	**0.912**	**0.951**	**0.950/0.949**	**0.905**	**0.950**	**0.667**	**0.583**	**0.593**	**0.493**
SegNet + Att^22^.	------	0.910	**0.955**	**0.954**/0.941	0.890	**0.953**	------	------	------	------
CNN + Att^53^.	------	0.860	0.949	0.943/0.853	0.745	0.936	------	------	------	------

## Supplement experiments and analysis results

We have added a supplement annotation consistency analysis using Cohen’s Kappa scores in Eq. 12 to validate a classifier against a baseline of random guessing, ensuring the model’s predictive power is genuine, which yields 0.6897 for the Thermal, 0.9626 for the PVEL-S, 0.7455 for the UCF-EL, and 0.8928 for the Benchmark EL datasets, demonstrating overall model reliability. These numbers indicate that the first and third datasets could need to be enhanced using different data augmentation methods, appropriate filters, or labelling methods such as adaptive soft labelling.

12 $$\:k=\frac{{P}_{o}-{P}_{e}}{1-{P}_{e}}$$

where P_e_ and P_o_ are the expected agreement (the probability of the model agreeing purely by chance, calculated using the proportion of times the model assigns each) and the observed agreement class (the actual percentage of times the model and the truth agree), respectively.

Supplemental relevant experiments are added to enhance the interpretability and reproducibility of the model design, which provide deeper insight into the architectural design choices. Due to computational constraints, ablation experiments were conducted on the PVEL-S and Benchmark EL datasets as representative cases. Table 12 shows hyperparameter sensitivity and architectural contribution analysis for each component individually.

**Table 12:** Ablation study results for model configuration of hyperparameter.

Model Configuration	PVEL dataset		Benchmark EL	
	Accuracy	mIoU	Accuracy	mIoU
Branches (B) = 1 have transformer layers (TL) = 3, heads (h) = 6, MLP= [64, 128], i/p depth = 256	0.9888	0.9594	0.9487	0.4637
B = 1: TL = 3, h = 6, MLP = [64, 128], depth = 512	0.9892	0.9608	0.9471	0.4552
B = 1: TL = 3, h = 2, MLP = [16, 32], depth = 512	0.9894	0.9615	0.9481	0.4586
B = 1: TL = 3, h = 6, MLP = [16, 32], depth = 512	0.9891	0.9603	0.9461	0.4515
B = 2: all of TL = 3, h = 6, [64, 128] MLP, 256 depth & h = 2, [16, 32] MLP, 512 depth	0.9897	0.9625	0.9464	0.4881
B = 2: all of TL = 3, h = 6, [64, 128] MLP, 256 depth & h = 4, [32, 64] MLP, 128 depth	0.9892	0.9606	0.9484	0.4727
B = 3 all of TL = 1	0.9895	0.9618	0.9459	0.4802
B = 3 all of TL = 2	0.9898	0.9628	0.9483	0.4897
B = 3 all of TL = 3, CNN layers without residuals	0.9893	0.9610	0.9432	0.4353
B = 3 all of TL = 3, up-sampling without skip connections	0.9892	0.9606	0.9444	0.4190
Proposed B = 3 all of TL = 3, with CNN residuals & skip connections	**0.9900**	**0.9636**	**0.9500**	**0.4926**

The ablation study confirms that the proposed configuration (B = 3, TL = 3, with residuals and skip connections) achieves the best performance on both datasets. Several observations emerge for PVEL-S binary dataset first, all configurations achieve high performance (> 98.88% accuracy, > 95.94% mIoU), confirming the model’s robustness to hyperparameter variations. Second, increasing the number of Transformer branches from 1 to 2 yields the largest gain (+ 0.09% accuracy, + 0.31% mIoU), while the third branch provides marginal additional improvement (+ 0.03% each). Third, adding Transformer layers from 1 to 3 progressively improves performance, with TL = 3 achieving the best results. Fourth, removing residuals or skip connections degrades performance by 0.07–0.08% in accuracy and 0.26–0.30% in mIoU, confirming their contributions. The effect on performance is not evident from increasing the number of heads and MLPs only; it seems to be affected more by i/p depth. Finally, the proposed configuration (three branches, three Transformer layers per branch, with residuals and skip connections) achieves the highest accuracy (99.00%) and mIoU (96.36%), outperforming all alternatives. The improvements are marginally but consistently outperforming all variants. The more challenging Benchmark EL multi-class dataset achieves 95.00% accuracy and 49.26% mIoU, improving upon the best single-branch baseline (94.87% accuracy, 46.37% mIoU) by + 0.13% accuracy and + 2.89% mIoU. Removing residuals or skip connections degrades performance on Benchmark EL substantially (mIoU drops by 5.7–7.4%), confirming their critical role. Given the extreme challenge of micro-defects in this dataset (per-class analysis shows sub-600px defects fail), these results confirm the optimality of our design. The small performance differences across configurations on PVEL-S (≤ 0.12% accuracy) further demonstrate model robustness.

To demonstrate the robustness and stability of the proposed model, we have conducted a 3-fold cross-validation method with approximate 40 epochs per fold (due to hardware limitation) using the training data of the four datasets. The thermal dataset gives a mean validation accuracy of 99.85% (individual folds: 99.86%, 99.84%, 99.86%) with standard deviation (std) ± 0.01% and a mean validation loss of 0.0263 ± 0.0069 also mean validation IoU of 71.74 ± 1.2623%. The PVEL-S dataset gives mean validation accuracy of 98.81% ± 0.06% (98.74%, 98.80%, 98.89%) and mean validation loss of 0.0298 ± 0.0016 also mean validation IoU of 95.54%±0.169%. The UCF-EL dataset mean accuracy of 95.95%±0.2095% (96.17%, 96.02%, 95.67%), mean loss 0.2384 ± 0.01736, and mean IoU of 52.85 ± 0.6719%. The Benchmark EL dataset gives an accuracy of 95.36% ± 0.16% (95.44%, 95.14%, 95.50%), loss of 0.1313 ± 0.0053, and IoU of 46.53% ± 0.99%. The overall 3-fold cross-validation across the four datasets in Table 13 reveals consistent patterns. The model achieves near-perfect stability on all datasets, as evidenced by low standard deviations in accuracy (≤ 0.21% for all). However, segmentation precision (IoU) varies substantially: PVEL-S shows excellent boundary alignment (95.54%), while Thermal (71.74%), UCF-EL (52.85%), and Benchmark EL (46.53%) reveal increasing challenges in defect boundary localization (due to minor PV defects or classes and data imbalance, especially for a large number of classes (29 in Benchmark EL)). This discrepancy between high accuracy and moderate IoU suggests that while the model reliably classifies defect regions, precise delineation of irregular defect boundaries remains the key bottleneck. The low cross-fold variance across all metrics (accuracy std < 0.21%, IoU std < 2% for most datasets) strongly confirms the model’s robustness and insensitivity to data partitioning.

**Table 13:** 3-fold cross-validation results.

Dataset	Mean Accuracy/std	Mean Loss/std	Mean IoU/std
Thermal	99.85%±0.01%	0.0263 ± 0.0069	71.74% ±1.2623%
PVEL-S	98.81% ± 0.06%	0.0298 ± 0.0016	95.54%±0.169%
UCF-EL	95.95%±0.2095%	0.2384 ± 0.01736	52.85%± 0.6719%
Benchmark EL	95.36% ± 0.16%	0.1313 ± 0.0053	46.53% ± 0.99%

Additional analysis based on detailed test results in Table 10 of the fourth Benchmark EL dataset to identify the reasons for the data’s challenges: we have analyzed the relationship between defect size and detection success using a size threshold. F1 vs. log pixel-count calculations reveal a clear threshold: all classes with > 10,000 pixels achieve F1 > 0.88, while all classes with < 600 pixels have F1 < 0.13 (mean F1 = 0.06 ± 0.06). Between 600 and 10,000 pixels, identifying the model’s effective receptive field limit. For failing classes, precision (P) exceeds recall (R) substantially: text (*P* = 0.36, *R* = 0.05), star (*P* = 0.33, *R* = 0.015), rings (*P* = 0.62, *R* = 0.07). This indicates the model can identify some tiny defect pixels only (a recall-dominated problem). Complete failures (crack rbn edge, dead cell with < 10 pixels) show P = *R*=0. The recall gap suggests that high-resolution features are lost in the encoder-decoder pathway. This directly motivates: (1) Feature Pyramid Networks to preserve spatial resolution via top-down pathways (create multi-scale feature pyramids with top-down pathways), and (2) Atrous Spatial Pyramid Pooling (ASPP) to expand receptive fields without reducing resolution as a future work (with various dilation rates to capture multi-scale context). (3) class-balanced sampling or focal loss to address extreme pixel imbalance (e.g., dead cell: 2 pixels vs. background: 1.29 M). Preliminary analysis suggests such modules could improve F1 on tiny defect classes from near-zero to 0.4–0.6. Performance analysis across all four datasets shows that the PVEL-S dataset achieves excellent and balanced performance across both classes (defect F1 = 0.968, non-defect F1 = 0.994), confirming strong generalization without class bias. The Thermal dataset has overall accuracy of 99.85%, but this is dominated by the background class (99.8% of pixels). The faulty cell class (5,064 pixels, 0.2% of dataset) achieves only F1 = 0.690, revealing that high accuracy masks moderate defect detection due to extreme class imbalance. That dataset would need one of the data augmentation methods to overcome this limitation. The Benchmark EL dataset shows a strong size-performance correlation (Pearson correlation coefficient (r) = 0.7123 with P-value = 6.4828 × 10⁻⁵ < 0.001). All defect classes with < 600 pixels have F1 < 0.13, including three classes with F1 = 0 with < 10 pixels. This systematic failure on tiny defects motivates multi-scale feature enhancement (e.g., FPN, ASPP). The UCF-EL has the poorest class performance for the Interconnect class (F1 = 0.232, 20,989 pixels), while similarly sized Corrosion class achieves F1 = 0.808. This indicates a semantic bottleneck (class distinguishability) rather than a size limitation, suggesting contrastive learning or refined class definitions as future solutions. While the model reveals three distinct bottlenecks on other datasets: class imbalance (Thermal), defect size (Benchmark EL), and class distinguishability (UCF-EL), it excels in PVEL-S dataset. These dataset-specific limitations provide clear, actionable pathways for future work.

In general, the proposed hybrid enhanced Transformer model outperforms existing methods, achieving high accuracy and good precision for PV failure localization across four diverse datasets, while revealing clear pathways for addressing remaining micro-defect challenges.

## Conclusion, recommendations and future works

This study introduces a new deep learning approach that presents a data-driven approach for PV fault localization and detection through image semantic segmentation. We developed and evaluated a hybrid Transformer neural network model with four different datasets. First, the thermal dataset was utilized to demonstrate large-scale PV plant inspection in detecting and locating failure cells, achieving exceptional accuracy of 99.89%. For cell-level fault analysis, electroluminescence (EL) images were employed for both binary and multi-class segmentation. The second dataset gives a superior result with 99% accuracy and 96.36% mIoU for locating the defective areas in the cell. For multiclass segmentation the third and fourth datasets are investigated with an accuracy of 96.27% and 95%, respectively, which demonstrates that our FDD and localization model can perform robustly across diverse datasets, demonstrating robust PV failure localization under various challenging conditions.

This research serves as a valuable reference for researchers and the PV industry to increase the likelihood of problem localization and detection in solar PV systems. We recommend evaluating the proposed methodology on additional large-scale datasets with various fault class segmentations to identify any potential limitations of our approach.

Future work should explore sophisticated data augmentation methods like instance-aware copy-paste or online hard-example mining augmentations to artificially increase the presence of rare defect patterns (like some classes in the fourth dataset) for improving its performance. Furthermore, the use of conditional GANs or latent diffusion models specifically designed for semantic segmentation in synthetic data generation enables the generation of realistic defect examples without artefacts. Also, we may explore adaptive patch partitioning, hybrid local-global attention reweighting or multi-scale feature enhancement (e.g., FPN, ASPP). These approaches may lead to better detection performance for the hardest minority classes than what is possible with only fine-tuning the loss function. Future work also will focus on developing a simple, lightweight real-time implementation in the field with the help of the Internet of Things (IoT) to help speed maintenance, like complementary knowledge distillation techniques, where the current model serves as a teacher to train a compact student network model (e.g., feature-based alignment or attention map transfer) or developing lightweight transformer versions (e.g., MobileViT, EfficientFormer, shifted window and linear attention). All are promising possibilities, evaluating trade-offs between segmentation accuracy, inference latency, and model size on embedded devices for real-time, large-scale smart PV monitoring. Additionally, examining diverse hybridization architectures utilizing the transformer neural network for the localization of small and minor faults and exploring full hyperparameter grid search and cross-dataset ablation for more reliability in future.

## Data Availability

The data used in this study are four different datasets:- The first **Photovoltaic Thermal Dataset** Images are available from the Universit`a Politecnica delle Marche - DII - VRAI Group repository at [http://vrai.dii.univpm.it/content/photovoltaic-thermal-images-dataset](http:/vrai.dii.univpm.it/content/photovoltaic-thermal-images-dataset) after completing the request form and contract conditions, in which the candidate (researcher with an email address affiliated with an institution or university) outlines their goals for the study. A download link and credentials are sent after the owners’ approval with the condition that they are for research purposes only, with no other use. Notice: “That data are not publicly redistributable and were used under license for the current study; however, by following the repository’s request procedure, the access to the data for replication or verification reasons can be acquired by a researcher.“- The second **PVEL-S Electroluminescence Dataset** Images are publicly available at [https://www.kaggle.com/datasets/yaozhang01182010/dataset-of-solar-cells-defect-segmentation](https:/www.kaggle.com/datasets/yaozhang01182010/dataset-of-solar-cells-defect-segmentation).- The third **UCF-EL Dataset** Images are publicly available at [https://github.com/ucf-photovoltaics/UCF-EL-Defect](https:/github.com/ucf-photovoltaics/UCF-EL-Defect).- The fourth **Benchmark EL Dataset** Images are publicly available at [https://github.com/TheMakiran/BenchmarkELimages.git](https:/github.com/TheMakiran/BenchmarkELimages.git).The generated data during the course of the research, as software algorithm code, is owned privately by the corresponding author, who can be contacted with any questions or concerns regarding the algorithm or data.
